# Akt-Signal Integration Is Involved in the Differentiation of Embryonal Carcinoma Cells

**DOI:** 10.1371/journal.pone.0064877

**Published:** 2013-06-06

**Authors:** Bo Chen, Zheng Xue, Guanghui Yang, Bingyang Shi, Ben Yang, Yuemin Yan, Xue Wang, Daishu Han, Yue Huang, Wenji Dong

**Affiliations:** 1 Department of Cell Biology, Institute of Basic Medical Sciences, Chinese Academy of Medical Sciences and Peking Union Medical College, Beijing, China; 2 Department of Biochemistry, Institute of Basic Medical Sciences, Chinese Academy of Medical Sciences and Peking Union Medical College, Beijing, China; 3 Department of Medical Genetics, Institute of Basic Medical Sciences, Chinese Academy of Medical Sciences and Peking Union Medical College, Beijing, China; 4 State Key Laboratory of Medical Molecular Biology, Institute of Basic Medical Sciences, Chinese Academy of Medical Sciences and Peking Union Medical College, Beijing, China; Baylor College of Medicine, United States of America

## Abstract

The mechanism by which Akt modulates stem cell homeostasis is still incompletely defined. Here we demonstrate that Akt phosphorylates special AT-rich sequences binding protein 1 (SATB1) at serine 47 and protects SATB1 from apoptotic cleavage. Meanwhile, Akt phosphorylates Oct4 at threonine 228 and Klf4 at threonine 399, and accelerates their degradation. Moreover, PI3K/Akt signaling enhances the binding of SATB1 to Sox2, thereby probably impairing the formation of Oct4/Sox2 regulatory complexes. During retinoic acid (RA)-induced differentiation of mouse F9 embryonal carcinoma cells (ECCs), the Akt activation profile as well as its substrate spectrum is strikingly correlated with the down-regulation of Oct4, Klf4 and Nanog, which suggests Akt activation is coupled to the onset of differentiation. Accordingly, Akt-mediated phosphorylation is crucial for the capability of SATB1 to repress Nanog expression and to activate transcription of *Bcl2* and *Nestin* genes. Taken together, we conclude that Akt is involved in the differentiation of ECCs through coordinated phosphorylations of pluripotency/differentiation factors.

## Introduction

Stem cells possess the properties of self-renewal and differentiation potential. Modulators of the PI3K/Akt signaling pathway including PTEN [1], [2], PML (promyelocytic leukemia) [3], TSC [4] and Fbxw7 [5], [6] and effectors including FoxO transcriptional factors [7], [8], [9] and p21Cip [10] are indispensible for the homeostasis of normal hematopoietic stem cells (HSCs), implying that abnormal activation of Akt negatively regulates HSC stemness. The functions of Akt in embryonic stem cells (ESCs) [11], adult stem cells [12] and cancer stem cells (CSCs) [8] have been investigated, but its precise role in the maintenance of stem cell homeostasis and the mechanism by which Akt modulates differentiation are yet to be clarified. Although common approaches such as forced gene expression, genetic knockdown and the use of pathway agonists/inhibitors all give clues as to the functions of Akt, these manipulations always lead to global and promiscuous effects. Therefore, identifying and characterizing novel substrates of Akt that are functionally related to pluripotency and are involved in the regulation of differentiation is a reasonable way to illustrate its functions.

The “core” transcriptional factors, including Oct4, Sox2 and Nanog, are of great importance to maintaining the stemness of ESCs [13]. Introduction of four reprogramming factors (Oct4, Sox2, Klf4 and c-Myc) reprograms mouse embryonic fibroblast cells into induced pluripotent stem cells (iPSCs) [14]. Thus it is apparent that these transcriptional factors play indispensable roles in the establishment and maintenance of pluripotency state. SATB1, a chromatin organizer and global gene regulator, represses expression of *Nanog* and *Klf4* in ESCs [15]. It also defines a differentiation context for gene silencing by Xist, a lncRNA which triggers the onset of X chromosome inactivation [16], although such an observation was challenged recently [17]. By contrast, Oct4, Sox2 and Nanog bind to intron 1 of Xist to suppress its expression in undifferentiated female ESCs [18]. Therefore, it is evident that SATB1 counteracts the roles of pluripotency factors during the onset of cell differentiation. Intriguingly, one common feature shared by SATB1, Oct4, Sox2 and Klf4 is that they all have a consensus Akt phosphorylation motif (RxRxxS/T) [19], [20], which raises the possibility that Akt is a master signaling molecule to modulate the antagonizing status between SATB1 and pluripotency factors.

In the present study, we focused on a number of pluripotency/differentiation-regulating factors that have potential and conserved Akt phosphorylation motifs. We identified several novel substrates of Akt by using *in vitro* kinase assay, including Oct4, Klf4, Bmi-1, MBD3, Twist1, Fbxw7 and SATB1. Based on preliminary data, considering the opponent effects of SATB1 on the expression of *Nanog* and *Klf4*, we proposed that Akt may phosphorylate the differentiation factor SATB1 and the pluripotency factors Oct4 and Klf4 simultaneously or sequentially to change their abundance or conformation, thereby dictating stem cells to maintain the pluripotency status or enter the differentiation program. Our data favors a model that Akt facilitates the differentiation process through coordinated regulation of SATB1 versus Oct4/Klf4, i.e. by boosting SATB1 function yet attenuating Oct4/Klf4 activity. This conclusion is consistent to the fundamental function of Akt as a survival kinase, whereby that activated Akt gives rise to more progenies of stem cells would result in a partial loss of stemness in a population level.

## Materials and Methods

### Reagents

The antibodies used in this study were anti-Myc and anti-Flag (Sigma); anti-phospho-serine/threonine (Qiagen); anti-HA (Roche); anti-Akt, anti-Akt1, anti-Akt2, anti-Akt3, anti-phospho-Akt (Thr-308), anti-phospho-Akt (Ser-473) and anti-phospho-Akt substrate (RxRxxS/T) (Cell Signaling Technology); anti-SATB1 (BD Biosciences, Cell Signaling Technology, Abcam); anti-GAPDH (Upstate Biotechnology, Inc); anti-Oct4, anti-Klf4, anti-Sox2, anti-Nanog, anti-actin and anti-Ubiquitin (Santa Cruz Biotechnology, Inc). Anti-rabbit, anti-mouse and anti-goat horseradish peroxidase (HRP) conjugated secondary antibodies were purchased from Vector Laboratories. AKTi-1/2, Wortmannin, IGF-1 (insulin-like growth factor 1), MG132, cycloheximide (CHX), camptothecin (CPT) and retinoic acid (RA) were purchased from Sigma. LY294002 was from CalBiochem. The peptide N-CAAARGRLG(pS)TGAKM-C was synthesized and anti-phospho-SATB1 (Ser-47) was raised in rabbits in Genemed Synthesis Inc. The peptide N-RKRKR(pT)SIEN-C was synthesized, and anti-phospho-Oct4 (Thr-228) was raised in rabbits in Medical and Biological Laboratories Co., Ltd.

### Plasmids and Site-directed Mutagenesis

Plasmids of pUSE-Akt1 (wild-type, WT), pUSE-MyrAkt1 (activated, N-terminal myristoylation, Myr) and pUSE-Akt1 K179M (dominant negative, DN) were purchased from Upstate Biotechnology, Inc. The orf of cDNA encoding genes that were used in this study was subcloned into the appropriate expression vectors. The constructs of p3xFlag/SATB1 and pGEX4T-1/MD+HD (MARs binding domain; homeodomain) were kindly gifted from Dr. Sanjeev Galande (National Center for Cell Sciences, India). The mutations on the corresponding cDNA were generated with QuikChange II Site-Directed Mutagenesis Kit (Stratagene) according to manufacturer’s protocol. Oligonucleotides used in this study were listed in Supplementary information (Text S1). All plasmids were verified by DNA sequencing.

### Cell Culture and Transfection

HEK293A, HEK293T, 293FT, MCF-7, F9 (Cell Resource Center, Institute of Basic Medical Sciences, CAMS/PUMC) and retrovirus packaging cell line Plat-GP (Cellbiolabs) were maintained in Dulbecco's modified Eagle's medium (HyClone) containing 10% heat-inactivated fetal bovine serum (FBS), penicillin (100 units/ml) and streptomycin (100 µg/ml) in a humidified incubator with 5% CO_2_ at 37°C. Breast cancer cell lines of SK-BR-3 and MDA-MB-231 (Cell Resource Center, Institute of Basic Medical Sciences, CAMS/PUMC) were maintained in RPMI1640 medium (HyClone) containing 10% FBS. Cell transfections were carried out with Lipofectamine 2000 reagent (Invitrogen) according to the manufacturer's instruction.

### Generation of Recombinant Virus and Stable Cell Lines

For recombinant retrovirus packaging, Plat-GP cells were transfected with recombinant retroviral vectors and pCMV-VSV-G. For recombinant lentivirus packaging, 293FT cells were transfected with lentiviral vectors, together with packaging plasmids psPAX2 and MD2.G. Recombinant viruses were collected, filtered through 0.45 µm membrane and utilized to transduce target cell lines. Breast cancer cell lines SK-BR-3 and MDA-MB-231 transduced with recombinant retrovirus were subjected to selection with antibiotics (500 µg/ml of G418 or 1 µg/ml of puromycin) and antibiotics-resistant clones were pooled serving as stable cell lines. F9 cells were transduced with recombinant lentivirus and subjected to puromycin selection. Expression of wild-type or mutant proteins was verified by Western blotting with appropriate antibodies.

### Recombinant Protein Production and Purification

Briefly, bacteria culture with *A*_600_ = 0.6 were induced with 0.4 mM of isopropyl-1-thio-β-galactopyranoside (IPTG) for 2 h at 30°C. The cells were pelleted by centrifugation at 13,000×g for 15 min at 4°C, and then resuspended in phosphate-buffered saline (PBS) containing 1% Triton X-100 and a protease inhibitor mixture (4 µg/ml aprotinin, 4 µg/ml leupeptin, 4 µg/ml antipain, 12.5 µg/ml chymostatin, 12 µg/ml pepstatin, 130 µg/ml ε-aminocaproic acid, 200 µg/ml *p*-aminobenzamidine and 1 mM phenylmethylsulfonyl fluoride). The cells were sonicated on ice and cell debris was then removed by centrifugation at 12,000 × *g* for 10 min at 4°C. The glutathione *S*-transferase (GST) fusion proteins were purified by glutathione-Sepharose affinity chromatography according to the manufacturer’s instructions for batch purification (GE Healthcare). The purity and the amount of fusion proteins were analyzed by SDS-PAGE.

### GST Pull-down Assay

HEK293T cells transfected with expression plasmid were gently washed one time with ambient PBS and lysed in l mL of modified RIPA buffer containing 50 mM Tris, pH 7.4, 150 mM NaCl, 1 mM EDTA, 1% Nonidet P-40, 0.25% Na-deoxycholate, 1 mM PMSF, 1 mM Na_3_VO4, 1 mM NaF and 1µg/ml each of Aprotinin, Leupeptin and Pepstatin at 4°C for 15 min. The cellular lysates were centrifuged at 13,000×g for 15 min at 4°C, and precleared by incubation with GST-glutathione-Sepharose beads for 30 min at 4°C. The glutathione-Sepharose beads, immobilized GST or GST fusion proteins, were incubated with precleared lysates at 4°C for 2 h, respectively. The beads were then centrifuged at 2,500 rpm for 5 min at 4°C, and washed twice with RIPA buffer and three times with PBS containing protease inhibitors. The proteins were released by addition of 40 µL of 2×SDS-PAGE loading buffer followed by boiling. The samples were then subjected to SDS-PAGE and immunoblotting.

### Immunoprecipitation

HEK293T cells cotransfected with plasmids coding different proteins were lysed at 4°C for 15 min in 1 ml of modified RIPA buffer. The cells were disrupted by repeated aspiration through a 21-gauge needle followed by a 26-gauge needle and the lysates were centrifuged at 13,000×g for 15 min at 4°C to remove the cellular debris. The cell lysates were pre-cleared by incubation with control IgG and protein G-agarose beads (Santa Cruz Biotechnology, Inc). The supernatant was incubated with appropriate antibody and protein G-agarose beads at 4°C overnight. The immunocomplex was pelleted by centrifugation at 2,500 rpm for 5 min at 4°C, and then was washed extensively with RIPA buffer. The immunoprecipitated proteins were eluted by boiling in SDS-PAGE buffer for 5 min and subjected to Western blotting with the appropriate antibodies.

### *In vitro* Kinase Assay

HEK293T cells transfected with plasmid of Myc-tagged Akt1 (Myr) were harvested and lysed in buffer A containing 50 mM Tris, pH 7.4, 1 mM EDTA, 1 mM EGTA, 1% Triton X-100, 50 mM NaF, 5 mM Sodium Pyrophosphate, 10 mM Sodium β-glycerophosphate, 0.1% (v/v) 2-mercaptoethanol, 1 mM PMSF, 1 mM Na_3_VO_4_ and 1 µg/ml each of Aprotinin, Leupeptin and Pepstatin. The cell lysates were pre-cleared by incubation with control IgG and protein G-agarose beads (Santa Cruz Biotechnology, Inc). The supernatant was incubated with anti-Myc and protein G-agarose beads at 4°C overnight. Immunocomplex was washed twice with buffer A supplemented with 500 mM NaCl, twice with buffer B containing 50 mM Tris, pH 7.4, 0.1 mM EGTA and 0.1% (v/v) 2-mercaptoethanol, and twice with kinase assay buffer (20 mM Tris-HCl, pH 7.5, 10 mM MgCl_2_, 5 mM DTT and 0.1 mM Na_3_VO_4_). The immunoprecipitated Akt1 was incubated with appropriate amount of GST fusion proteins in 40 µl of kinase assay buffer containing 200 µM ATP and 5 µCi [γ-^32^P] ATP (3000 Ci/mmol) (PerkinElmer Life Sciences) for 60 min at 30°C. At the end of the reaction period, the reaction was ceased by adding 40 µl of 2× SDS-PAGE loading buffer and boiling for 5 min. The reaction mixtures were resolved on 10% SDS-PAGE and were then subjected to autoradiography or immunoblotting.

### SDS-PAGE and Western Blot Analysis

Cell lysates and immunoprecipitates were resolved by 10% SDS-PAGE and transferred to polyvinylidene difluoride membranes. The blots were blocked for 60 min in 1×PBS containing 5% skimmed milk and 0.1% Tween-20, probed with the appropriate primary antibodies in the same blocking solution. The secondary antibody was a horse anti-rabbit or a horse anti-mouse IgG HRP-conjugated antibody (Vector laboratories) diluted 5000-fold in the blocking buffer. The blots were developed by the enhanced chemiluminescence (Amersham Biosciences).

### Fluorescence Microscopy

HEK293A cells grown on glass coverslips were transfected with either plasmids encoding GFP-SATB1 together with Myc-Akt1 WT, Myc-Akt1 Myr or Myc-Akt1 DN, or plasmids of GFP alone, GFP-SATB1, GFP-SATB1S47A or GFP-SATB1S47D using Lipofectamine 2000 (Invitrogen). Transfected cells were cultured for 24 h. Fluorescent cells were viewed using fluorescence microscopy, and images were aquired with a CCD camera.

### RNA Isolation and Analysis

Total RNA was isolated by using Trizol (Invitrogen). For real-time PCR analysis, cDNA was synthesized from total RNA by M-MuLV reverse transcriptase (New England Biolabs) with random primers (Takara). The resulting cDNA was subjected to PCR analysis with gene-specific primers, using IQ5 Realtime PCR system (Bio-Rad). The PCR product was measured by SYBR green (Takara).

### Chromatin Immunoprecipitation (ChIP) Assay

The F9 stable cell lines carrying empty vector, wild-type SATB1, SATB1S47A or SATB1S47D were cross-linked with 1% formaldehyde for 10 min at room temperature. Glycine was added to a final concentration of 125 mM and cells are incubated on ice for 5 min to stop cross-linking. Cells were then washed three times with ice-cold PBS and resuspended in hypotonic buffer (10 mM HEPES, pH 7.9, 1.5 mM MgCl_2_, 10 mM KCl, 0.5% NP-40 with protease inhibitors freshly added). Nuclei were spun down after 30 min incubation on ice, resuspended in sonication buffer (50 mM HEPES, pH 7.9, 1 mM EDTA, pH 8.0, 140 mM NaCl, 0.1% SDS, 1% Triton X-100, 0.1% sodium deoxycholate with protease inhibitors freshly added) and sonicated to obtain DNA fragments with an average size of 500 base pairs. Sonicated chromatin was diluted in dilution buffer (16.7 mM Tris, pH 8.1, 1.2 mM EDTA, pH 8.0, 167 mM NaCl, 0.01% SDS, 1.1% Triton X-100). For chromatin from 3×10^7^ cells, 80µl of anti-FLAG M2 affinity gel (Sigma F2426, precleared with 50µg of sheared salmon sperm DNA and 100µg of bovine serum albumin) was added and incubated overnight. As the negative control, 0.1 mg/ml 3×FLAG peptide (Sigma F4799) was added along with M2 affinity gel to block its binding to the FLAG epitope. Beads were then washed one time with low immune complex wash buffer (20 mM Tris, pH 8.1, 2 mM EDTA, 150 mM NaCl, 0.1% SDS, 1% Triton X-100), one time with high immune complex wash buffer (20 mM Tris, pH 8.1, 2 mM EDTA, 500 mM NaCl, 0.1% SDS, 1% Triton X-100), one time with LiCl immune complex wash buffer (10 mM Tris, pH 8.1, 1 mM EDTA, 0.25 M LiCl, 1% NP-40, 1% Sodium deoxycholate), two times with TE (10 mM Tris, pH 8.0, 1 mM EDTA). Beads were eluted twice for 1 h at 4°C using 200µl of 0.1 mg/ml 3×FLAG peptide in PBS. The elution was used to extract DNA for quantitative PCR.

## Results

The observation that Akt is activated during the stage switch from quiescent to active adult stem cells raises the question of whether Akt regulates self-renewal through control of its downstream stemness/differentiation substrates. We chose a number of candidate Akt substrates according to the following criteria: (1) they modulate stemness/differentiation; (2) they have the conserved Akt phosphorylation motif RxRxxS/T. We carried out an *in vitro* kinase assay to address whether Akt could directly phosphorylate these candidate substrates (Figure S1). We checked the phosphorylation by Akt using phospho-Akt substrate antibody and autoradiography. Positive signals from both antibody detection and the autoradiography were set up as golden standard for further characterization. An autoradiographic signal alone was a less stringent standard if anti-phospho-Akt substrate antibody failed to recognize the candidate motifs. We regarded antibody detection reliable when a positive signal was detected in the presence of ATP and no band presented with a mutant GST fusion protein as a substrate. We identified several novel substrates of Akt, including SATB1, Oct4 and Klf4 (Table S1). It is known that SATB1 transcriptionally represses the expression of *Klf4* and *Nanog,* we therefore hypothesized that Akt signaling is probably involved in regulation of stemness through phosphorylation of SATB1, Oct4 and Klf4 and by enhancing the antagonistic role of SATB1 on Nanog and Klf4.

### Akt Phosphorylates SATB1

**Figure 1:**
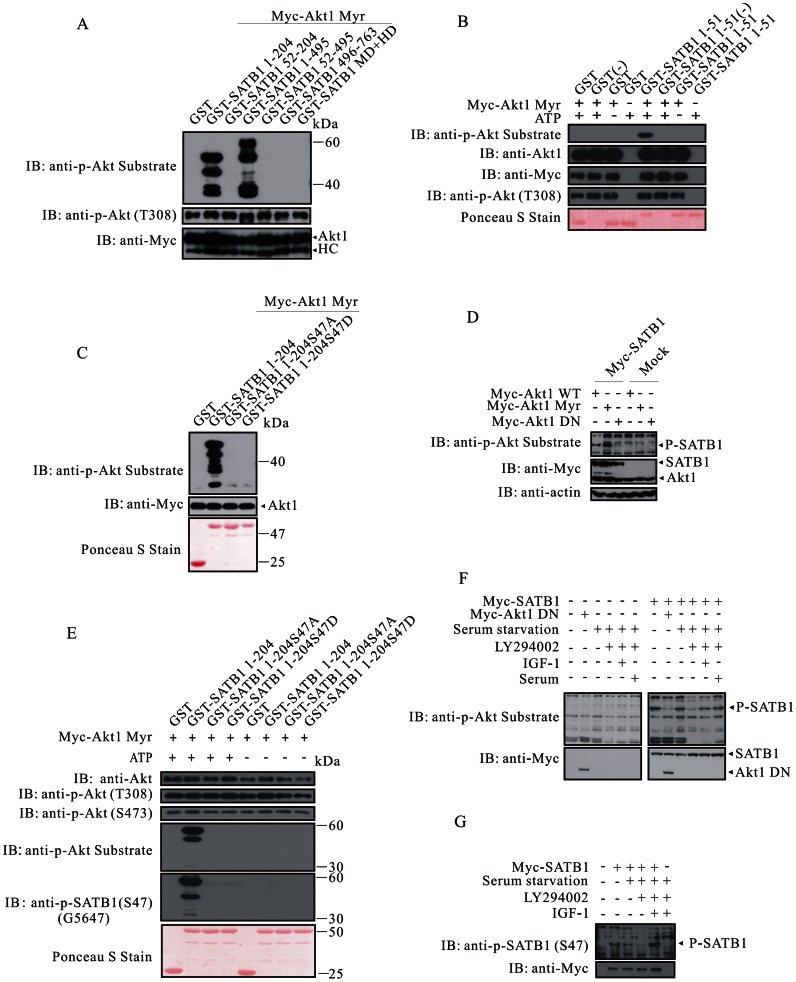
Akt phosphorylates SATB1 at serine 47 in a PI3K-dependent manner. (**A**) Akt phosphorylates GST-SATB1 1–204 and GST-SATB1 1-495. Purified GST fusion proteins were subjected to *in vitro* kinase assay in the presence of immunoprecipitated Akt. The samples were resolved on 10% SDS-PAGE and blotted with anti-phospho-Akt substrate, anti-phospho-Akt (T308) and anti-Myc. (**B**) The phosphorylation site resides on SATB1 within an N-terminal fragment spanning amino acids 1–51. (**C**) Akt phosphorylates GST-SATB1, but not GST-SATB1 S47A or GST-SATB1 S47D. (**D**) Ectopically expressed Myc-SATB1 is phosphorylated by Akt. Myc-SATB1 was co-expressed with Myc-Akt (WT), Myc-Akt (Myr) or Myc-Akt (DN) in HEK293T cells. Whole-cell lysates were subjected to immunoblotting with anti-phospho-Akt substrate, anti-Myc and anti-Actin. (**E**) The anti-phospho-SATB1 (S47) raised in rabbit specifically recognizes the phosphorylated SATB1 at serine 47. (**F**) HEK293T cells were transfected with empty vector or Myc-SATB1, 24 h post-transfection, cells were rinsed twice with PBS and replenished with fresh DMEM with no FBS supplemented. After additional 24 h, cells were treated with LY294002 (20 µM) for 2 h, followed by 20 min stimuli with or without IGF-1 (50 ng/ml) or FBS (20%). Whole-cell lysates were subjected to western blotting. (**G**) HEK293T cells were treated as indicated. Whole cell lysates were subjected to western blotting with antibodies of anti-phospho-SATB1 (S47) and anti-Myc. HC, heavy chain; WCL, whole cell lysate; Myr, myristoylation.

SATB1 has a sequence motif (R^42^GRLGS^47^) that is highly conserved across species from fish to human (Figure S2A). Akt phosphorylated GST-SATB1 1–204 and GST-SATB1 1–495, rather than GST-SATB1 52–204 and GST-SATB1 52–495, which suggested the phosphorylation site resided within SATB1 1–52 (Figure 1A, Figures S2B and S2C). A shorter fusion protein GST-SATB1 1–51 further corroborated the notion that the phosphorylation site located within this region (Figure 1B). We next generated the mutant proteins and examined whether Akt phosphorylated SATB1 at serine 47 *in vitro*. GST-SATB1 1–204, but not GST, GST-SATB1 1-204S47A or GST-SATB1 1-204S47D, was efficiently phosphorylated by Akt (Figure 1C, Figures S1B and S2D). We then investigated whether Akt could phosphorylate SATB1 *in vivo*. To this purpose, we expressed Myc-tagged SATB1 together with either Myc-tagged wild-type Akt, Myc-tagged constitutively activated Akt or Myc-tagged kinase dead Akt in HEK293T cells (Figure 1D, Figure S2E). SATB1 did exist in a pool of Akt substrates reciprocally immunoprecipitated with phosphorylated Akt substrate antibody (Figures S2F, S2G and S2H). In order to unequivocally define SATB1 phosphorylation at serine 47 by Akt, we raised a polyclonal antibody G5647 against phosphorylated peptide of N-CAAARGRLG(pS)TGAKM-C in rabbit (Figure S3). In the presence of ATP, Akt phosphorylated GST-SATB1 1–204, but not GST, GST-SATB1 1-204S47A or GST-SATB1 1-204S47D when assayed with the anti-serum of G5647. Without ATP, GST-SATB1 1–204 could not be recognized by G5647, which clearly proved that serine 47 was the phosphorylation site of Akt (Figure 1E).

As Akt is a potent downstream effector of PI3K signaling pathway, we then ask whether SATB1 phosphorylation by Akt would rely on PI3K activation. Treatment with LY294002, a PI3K inhibitor, suppressed Akt activation, thereby abrogating SATB1 phosphorylation. Addition of IGF-1 or FBS (fetal bovine serum) following LY294002 treatment reactivated PI3K/Akt signaling, which in turn resulted in SATB1 phosphorylation (Figure 1F). As a control, SATB1 phosphorylation was increased by constitutively activated Akt compared with wild-type Akt, whereas it was significantly decreased in the presence of an epitopically expressed kinase dead Akt, a dominant-negative form lacking kinase activity (Figure 1F). We then confirmed that SATB1 phosphorylation at serine 47 relied on IGF1/PI3K/Akt signaling with the anti-phospho-SATB1 (S47) (Figure 1G). Collectively, the data supports the idea that PI3K/Akt pathway is required for SATB1 phosphorylation at serine 47.

### Akt Maintains Molecular Integrity of SATB1 through Phosphorylation at Serine 47

**Figure 2:**
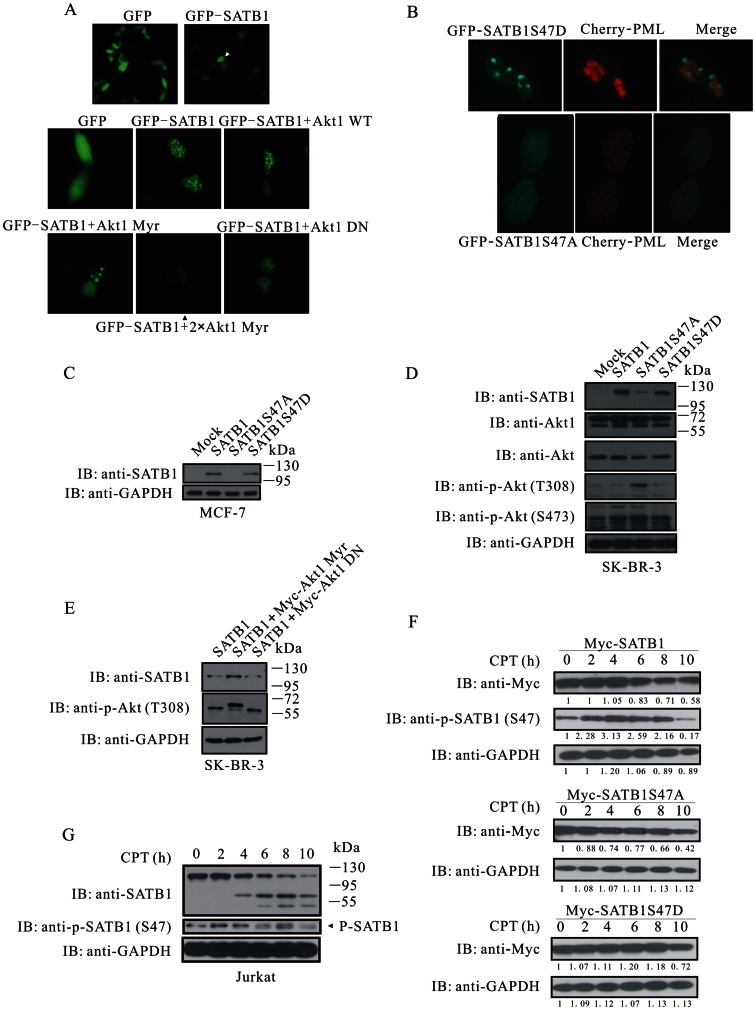
Akt shields SATB1 from apoptotic cleavage. (**A**) Evenly distributed signals were observed in the majority of HEK293A cells transfected with GFP-SATB1, whereas punctate structures were detected in a small fraction of cells (white arrowhead). Akt (Myr) decreased the ratio of cells with dot-like signals. (**B**) In cells with dot-like structures, GFP-SATB1S47A colocalized with Cherry-PML, whereas GFP-SATB1S47D did not. (**C**) Expression of SATB1 was analyzed in MCF-7 cells stably integrated with empty vector, wild-type SATB1, SATB1S47A or SATB1S47D, respectively. (**D**) Expression of SATB1 and Akt activation were analyzed in stable SK-BR-3 cell lines. (**E**) SATB1 stability and Akt activation were documented in SK-BR-3 cells carrying wild-type SATB1 together with Akt (Myr) or Akt (DN). (**F**) HEK293A cells were transfected with Myc-SATB1, Myc-SATB1S47A or Myc-SATB1S47D, respectively, and treated with CPT for 0, 2, 4, 6, 8 or 10 h. The cell lysates were subjected to immunobloting with anti-SATB1, anti-phospho-SATB1 (S47) and anti-GAPDH. (**G**) Jurkat cells were treated with CPT as in (F). The cell lysates were subjected to immunoblotting with anti-SATB1, anti-phospho-SATB1 (S47) and anti-GAPDH.

A number of Akt substrates are relocated within cells upon phosphorylation by Akt, for instance, FOXO transcription factors [21] and p27Kip1 [22]. The finding that SATB1 is a nuclear protein and a novel Akt substrate as well leads us to test whether PI3K/Akt signaling is obligate to redirect SATB1 to cytoplasm or sub-nuclear structures. GFP-tagged SATB1 was localized in nucleus in transfected HEK293A cells with less than 10% cells having punctate structures (Figure 2A). Although co-expression of wild-type Akt marginally reduced the ratio of dot-like cells, increased doses of activated Akt gradually and markedly reduced the number of dot-like cells and the number of evenly distributed dots. Intriguingly, with the increased doses of activated Akt, the dots would aggregate to form one or two larger bodies. By contrast, dominant-negative Akt generated a pattern of more, much tinier dots (Figure 2A). This observation suggests that Akt could modulate intra-nuclear aggregation of SATB1.

It has been reported that SATB1 partially co-localizes with PML in PML nuclear bodies, whereby SUMOylated SATB1 is cleaved by caspase-6 [23]. To examine whether SATB1 phosphorylation by Akt would affect co-localization of SATB1 and PML, GFP-SATB1, GFP-SATB1S47A or GFP-SATB1S47D along with Cherry-PML was transfected into HEK293A cells. We observed those cells with punctate structure of SATB1 and found that GFP-SATB1S47A partially co-localized with PML, whereas GFP-SATB1S47D resided exclusively outside of PML nuclear body (Figure 2B). This result reveals that phosphorylated SATB1 at serine 47 escapes from being targeted for PML nuclear body and possible subsequent cleavage.

In order to analyze the effect of phosphorylation on SATB1 stability, we established breast cancer cell lines carrying empty vector, wild-type SATB1, SATB1S47A or SATB1S47D. Like mock transduction, the SATB1S47A mutant almost could not be detected in stable MCF-7 cells with SATB1 antibody (Figure 2C). Consistently, SATB1S47A was expressed at a much lower level in SK-BR-3 cells, compared with SATB1 and SATB1S47D (Figure 2D). Paradoxically, in cells with the SATB1S47A mutant, Akt was highly activated through an unknown mechanism (Figure 2D). Furthermore, the myristoylated Akt increased the amount of SATB1 over-expressed in SK-BR-3 cells, whereas the dominant-negative Akt did not (Figure 2E). These findings allow us to conclude that Akt maintains the molecular integrity of SATB1 through phosphorylation.

Besides lamin B, SAF-A/hnRNP-U and NuMA, SATB1 is a fourth nuclear matrix-binding molecule that is cleaved for degradation by caspase upon apoptotic stimuli [23], [24]. We next tested whether Akt phosphorylation would protect SATB1 from apoptotic cleavage. The SATB1S47D mutant was more stable than both SATB1 and SATB1S47A in response to camptothecin (CPT) treatment. The CPT treatment led to a gradual loss of pan-SATB1; however, the phosphorylated SATB1 reached a peak 4 h post-treatment and then declined (Figure 2F). Consistently, endogenous SATB1 in Jurkat cells manifested the same behavior upon CPT treatment (Figure 2G). These results suggest that apoptotic signal may transiently induce an increase of SATB1 phosphorylation, which in turn improves its resistance to apoptosis.

### Akt Associates with Oct4 and Accelerates Oct4 Degradation through Phosphorylation

**Figure 3:**
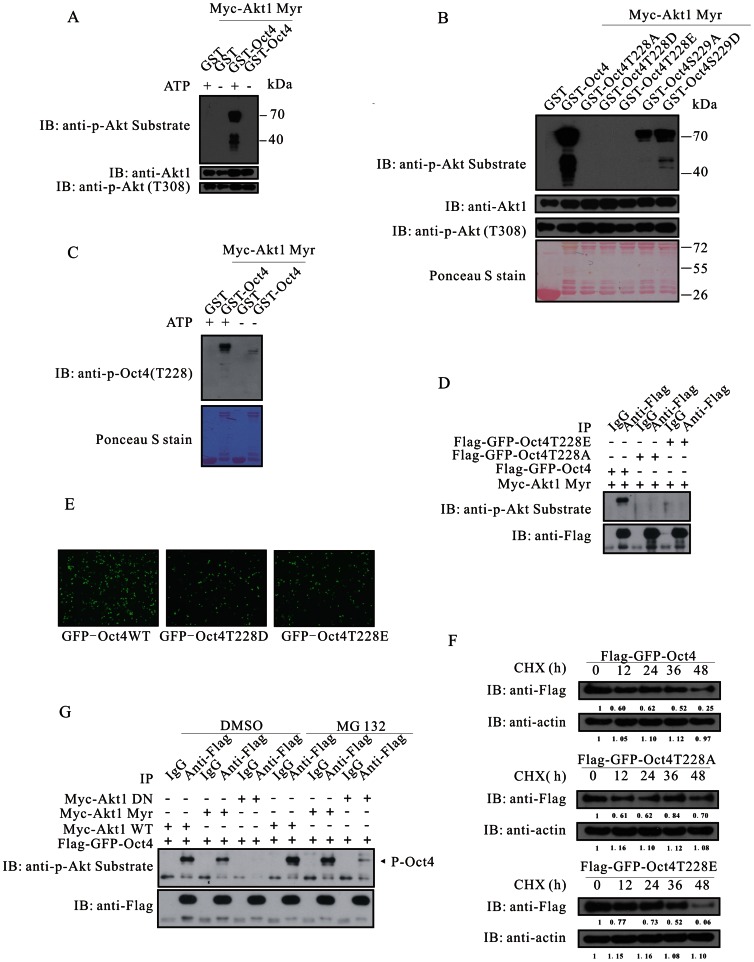
Akt phosphorylates Oct4 and accelerates its degradation. (**A**) Oct4 phosphorylation by Akt is ATP-dependent. (**B**) Akt phosphorylates GST-Oct4, but not GST, GST-Oct4T228A, GST-Oct4T228D or GST-Oct4T228E. Under the same reaction condition, mutation of serine 229 to alanine or aspartic acid significantly reduced the phosphorylation efficiency. (**C**) The anti-phospho-Oct4 antibody (T228) was raised in rabbit and its specificity was tested using *in vitro* kinase assay. (**D**) Akt phosphorylates wild-type Oct4, rather than its mutants at threonine 228. Flag-GFP-Oct4 or its mutants was ectopically expressed together with Myc-Akt (Myr) in HEK293T cells. Immunoprecipitates with anti-Flag were subjected to immunoblotting with anti-phospho-Akt substrate. (**E**) Expression of GFP-tagged Oct4 and its mutants in HEK293A cells. (**F**) Half-life of Oct4 and its mutants in transfected HEK293A cells treated with CHX (15 µg/ml). (**G**) HEK293A cells were transfected with Flag-GFP-Oct4 together with Myc-Akt (WT), Myc-Akt (Myr) or Myc-Akt (DN) and were treated with DMSO or MG132 (20 µM) for 5 h. Immunoprecipitates with anti-Flag were subjected to immunoblotting with anti-phospho-Akt substrate and anti-Flag.

The putative Akt phosphorylation site on Oct4 resides within a motif of (R^223^KRKRT^228^). *In vitro* kinase assay indicated that Akt phosphorylated Oct4 in an ATP-dependent manner (Figure 3A). A series of truncated GST-Oct4 constructs was made and GST fusion proteins were subjected to *in vitro* kinase assay (Figure S4A). Only GST fusion proteins that carried the putative Akt phosphorylation motif could be phosphorylated by Akt (Figure S4B). Oct4 has been shown to be phosphorylated at serine 229 by PKA [25]. The serine 229 is adjacent to the Akt phosphorylation motif, mutation of which may influence the phosphorylation efficiency of Akt. We therefore generated GST-Oct4 proteins with threonine 228 or serine 229 being mutated. Akt phosphorylated GST-Oct4, but not GST, GST-Oct4T228A, GST-Oct4T228D or GST-Oct4T228E (Figure 3B). As expected, the serine 229 mutations significantly decreased the reaction efficiency (Figure 3B). An antibody against phosphorylated Oct4 at threonine 228 was raised and purified. Using this antibody, we further confirmed Akt phsophorylated GST-Oct4 *in vitro* (Figure 3C). To examine whether Akt phosphorylated Oct4 *in vivo*, HEK293A cells were transfected with Myc-Akt (Myr) together with Flag-GFP-Oct4, Flag-GFP-Oct4T228A or Flag-GFP-Oct4T228E. Immunoprecipitation with anti-Flag followed by immunoblotting with anti-phospho-Akt substrate indicated that mutation of threonine 228 abolished phosphorylation-specific antibody recognition (Figure 3D). The GFP-tagged phosphorylation-mimetic mutants of T228D and T228E always manifested low GFP intensity and low GFP-positive cell number, which implied that phosphorylation might confer Oct4 unstable (Figure 3E). We therefore evaluated the effects of T228 mutations on Oct4 half-life. As illustrated in Figure 3F, compared with wild-type Oct4, T228A mutation extended the half-life of Oct4, whereas T228E mutation significantly shortened in transfected 293A cells treated with cycloheximide (CHX). To confirm that phosphorylated Oct4 was prone to be ubiquitinated and degraded, we treated cells with MG132, an inhibitor of 26S proteasome, and found that the amount of phosphorylated Oct4 was dramatically increased (Figure 3G, Figure S4D). We subsequently asked whether Akt associated with Oct4. To this purpose, a reciprocal co-immunoprecipitation indicated Akt interacted with Oct4 in an ectopically expressed system (Figure S4E and S4F). A reciprocal GST pull-down assay showed GST-Oct4 interacted with Akt, and GST-Akt interacted with Oct4 (Figure S4G and S4H). Importantly, endogenous Akt associated with Oct4 in mouse ES cells (Figure S4I and S4J). Collectively, these data indicate that Akt phosphorylates Oct4 and accelerates its degradation.

### Klf4 is an Akt Substrate

**Figure 4:**
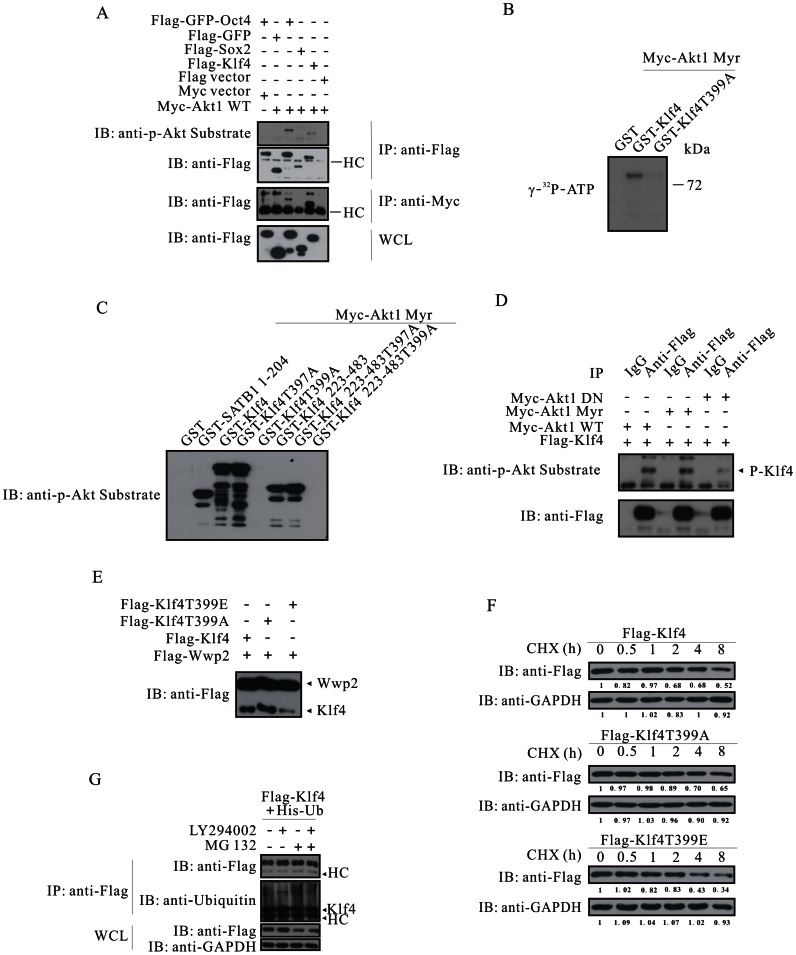
Akt phosphorylates Klf4 at threonine 399 and increases its degradation. (**A**) Akt associates with and phosphorylates Klf4. Reciprocal immunoprecipitations were carried out in HEK293A cells transfected as indicated. (**B and C**) Akt phosphorylates Klf4 at threonine 399, rather than threonine 397. (**D**) Akt phosphorylates Flag-Klf4 in transfected HEK293A cells. Immunoprecipitates with anti-Flag were subjected to immunoblotting with anti-phospho-Akt substrate and anti-Flag. (**E**) Klf4T399E is poorly expressed in transiently transfected HEK293A cells. Flag-Klf4, Flag-Klf4T399A or Flag-Klf4T399E was transfected together with Flag-Wwp2. Cell lystaes were subjected to immunoblotting with anti-Flag. (**F**) Half-life of Flag-Klf4 and its mutants in transfected HEK293A cells treated with CHX (15 µg/ml). (**G**) Inhibition of Akt activation by PI3K inhibitor LY294002 reduces Klf4 ubiquitination. Flag-tagged Klf4 and His-tagged Ubiquitin were co-expressed in HEK293A cells. Cells were serum starved and treated with LY294002 (20 µM) for 5 h, followed by 5 h with or without MG132 (20 µM). Immunoprecipitates with anti-Flag were subjected to immunoblotting with anti-Flag and anti-Ubiquitin. WCL, whole cell lysates.

Like Oct4 and Sox2, Klf4 also possesses a consensus Akt phosphorylation site within a motif of R^394^KRTAT^399^. The finding that Akt phosphorylates Oct4 prompts us to check whether Klf4 is also an Akt substrate. To this aim, we co-expressed Flag-GFP-Oct4, Flag-Sox2 or Flag-Klf4 together with Myc-Akt in HEK293A cells. Co-immunoprecipitation showed that Oct4 and Klf4, but not Sox2, associated with Akt. Akt phosphorylated Oct4 and Klf4, but not Sox2, as detected with anti-phospho-Akt substrate (Figure 4A). We then mutated threonine 399 to alanine and carried out *in vitro* kinase assay. Autoradiography indicated Akt phosphorylated GST-Klf4; however, under the same condition, Akt failed to modify GST or GST-Klf4T399A (Figure 4B). To exclude the possibility that threonine 397 was phosphorylated by Akt, using GST as a negative control and GST-SATB1 1–204 a positive control, we found that Akt phosphorylated threonine 399, rather than threonine 397 on Klf4 (Figure 4C). Immunoprecipitation with anti-Flag followed by immunoblotting with anti-phospho-Akt substrate revealed that Akt phosphorylated Klf4, and that activated Akt increased the phosphorylation signal, whereas dominant-negative Akt decreased the signal (Figure 4D). Although Wwp2 and Trim24, two E3 ubiquitin ligases, seemed to promote degradation of Oct4 and Sox2, both of them did not affect the level of Klf4 (Figure S4C). Using Flag-Wwp2 as a transfection control, we found that the expression level of the mutant T399A was higher than that of Klf4 without mutation; whereas the expression level of the mutant T399E was much lower (Figure 4E). Like Oct4, in comparison with wild-type and the T399A mutant, the mutation T399E significantly shortened the half-life of Klf4 in response to CHX treatment (Figure 4F). HEK293A cells were transfected with Flag-tagged Klf4 and His-tagged Ubiquitin, serum starved and treated with or without LY294002 and/or MG132. LY294002 treatment reduced the amount of ubiquitinated Klf4 (lane 1 versus lane 2), whereas MG132 treatment significantly increased ubiquitinated Klf4 (lane 1 versus lane 3), which suggested that PI3K/Akt signaling was required for ubiquitination and degradation of Klf4 (Figure 4G). In agreement to this observation, combined treatment with LY294002 and MG132 indicated that LY294002 compromised the effect of MG132 compared to treatment with MG132 alone (lane 3 versus lane 4) (Figure 4G). Taken together, these results reveal that Akt phosphorylates Klf4 at threonine 399, promoting Klf4 ubiquitination and degradation.

### Akt-mediated Phosphorylation Enhances SATB1 Binding to Sox2

**Figure 5:**
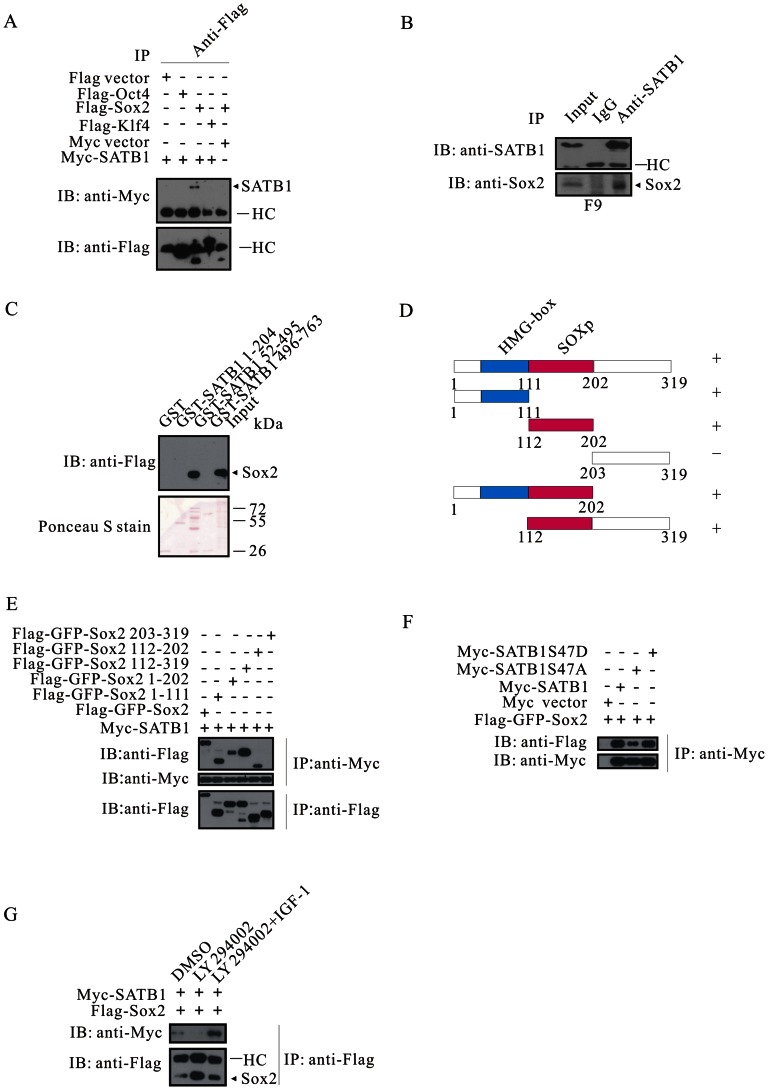
SATB1 binding to Sox2 is enhanced by Akt signaling. (**A**) SATB1 associates with Sox2, but not Oct4 or Klf4. (**B**) Endogenous interaction between SATB1 and Sox2. SATB1 was immuprecipitated from F9 cell lysates, immuprecipitations were subjected to immunblotting with anti-SATB1 and anti-Sox2. (**C**) Mapping of SATB1 domain required for Sox2 binding using GST pull-down assay. (**D**) A schematic representation of Flag-GFP-tagged Sox2 constructs is shown. (**E**) Mapping of Sox2 domains that are necessary for SATB1 binding. (**F**) SATB1S47A sequesters much less Sox2 than wild-type SATB1 and SATB1S47D. (**G**) Inhibition of Akt signaling disrupts SATB1/Sox2 interaction. HEK293T cells were co-transfected with Myc-SATB1 and Flag-Sox2, serum-starved and treated as indicated.

The above data indicates that Akt protects SATB1 from apoptotic cleavage; on the contrary, Akt phosphorylates Oct4 and Klf4, thereby promoting their degradation. It has been reported that SATB1 transcriptionally silenced expression of *Klf4* and *Nanog* in ESCs [15]. We next intend to understand whether there is an antagonistic effect between SATB1 and Oct4/Sox2/Klf4 at protein-protein interaction level. To answer this question, we co-expressed Myc-SATB1 together with Flag-Oct4, Flag-Sox2 or Flag-Klf4, respectively. Co-immunoprecipitation revealed that SATB1 interacted solely with Sox2 (Figure 5A). In addition, SATB1 endogenously associated with Sox2 in mouse F9 embryonal carcinoma cells (Figure 5B). We then mapped the interaction domains that were required for these two molecules. As shown in Figure 5C, Sox2 did not bind GST, GST-SATB1 1–204 or GST-SATB1 496–763, but solely bound GST-SATB1 52–495, implying that the region required on SATB1 was within a fragment spanning amino acids 204–495, which included domains of NMTS (nuclear matrix targeting sequence) and MD (MAR-binding domain). We subsequently made truncated Flag-GFP-tagged Sox2 constructs to determine which domain was necessary for SATB1/Sox2 association (Figure 5D). Except for the fragment of Sox2 203–319, SATB1 associated with the full-length Sox2, subdomains of 1–111, 1–202, 112–202 and 112–319 as well (Figure 5E, summarized at the far right of Figure 5D). These results suggest that both domains of 1–111 and 112–202 could independently mediate interaction with SATB1.

We then checked whether Akt signaling would influence the interaction between SATB1 and Sox2. Myc-tagged SATB1 or its phosphorylation-mimetic mutants was co-expressed with Flag-GFP-tagged Sox2 in 293A cells, respectively. Myc-SATB1S47A coimmunoprecipitated less Sox2 than Myc-SATB1 and Myc-SATB1S47D (Figure 5E). To confirm this observation, 293A cells were co-transfected with Myc-SATB1 and Flag-Sox2, serum starved and treated with DMSO, LY294002 or LY294002 plus IGF-1. Inhibition with LY294002 almost diminished the association between SATB1 and Sox2, compared to DMSO treatment. By contrast, addition of IGF-1 to LY294002 treated cells, which would reactivate Akt signaling, faithfully recapitulated SATB1/Sox2 interaction (Figure 5F). These results demonstrate that the Akt signaling obviously regulates the association between SATB1 and Sox2, probably contributing to Sox2 sequestration by SATB1 from an Oct4/Sox2 core transcription factor complex.

### Akt-mediated Phosphorylation is Crucial for the Role of SATB1 to Down-regulate the Nanog Expression during RA-induced F9 Cell Differentiation

Akt is significantly activated in cycling, rather than in quiescent stem cells [26]. Several modulators and effectors that antagonize the functions of Akt signaling have been demonstrated to be required for the maintenance of HSCs pool [27]. These studies altogether imply that Akt probably has a crucial role in the regulation of stem cell homeostasis. Using biochemical approaches, we identify three novel substrates of Akt that are functionally involved in pluripotency/differentiation regulation. Akt phosphorylates SATB1, a differentiation regulator for ESC, keeps it intact through apoptotic resistance, whereas Akt phosphorylates Oct4 and Klf4, two pluripoptency factors, accelerates their degradation, suggesting that Akt is a key upstream molecule that contributes to the differentiation program via inactivation of stemness transcriptional factors and/or activation of differentiation-regulating proteins.

**Figure 6:**
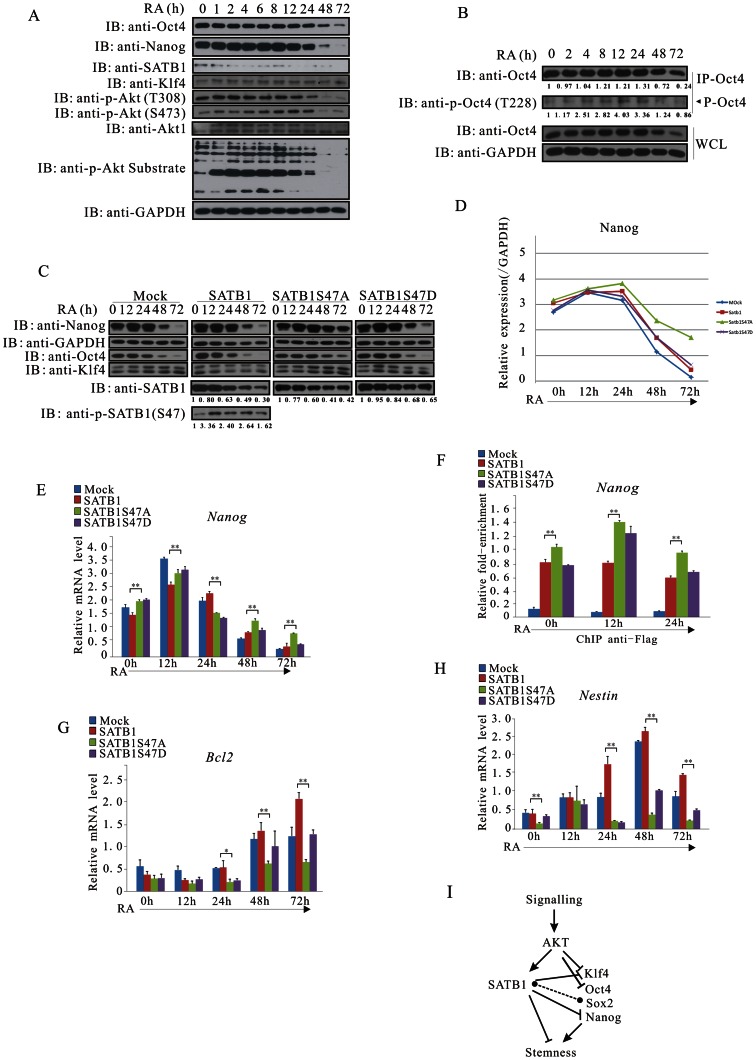
Akt-mediated phosphorylation is critical for the regulatory role of SATB1 in F9 cell differentiation and Nanog expression. (**A**) The profiles of Akt activity and its substrates match the change of pluripotency factors during RA-induced F9 cell differentiation process. F9 cells were seeded on petri dishes, induced with RA (1 µM) and harvested at 0, 1, 2, 4, 6, 8, 12, 24, 48 or 72 h. Cell lysates were subjected to immunoblotting with antibodies of anti-Oct4, anti-Nanog, anti-SATB1, anti-Klf4, anti-Akt1, anti-phospho-Akt (S473), anti-phospho-Akt (T308), anti-phospho-Akt substrate and anti-GAPDH. (**B**) The F9 stable cell lines were induced in the presence of RA as in (A) and harvested at 0, 2, 4, 8, 12, 24, 48 or 72 h, Immunoprecipitates with anti-Oct4 were subjected to immunoblotting with anti-Oct4 and anti-phospho-Oct4 (T228). (**C**) SATB1 and SATB1S47D are more efficient than SATB1S47A with respect to Nanog repression. The F9 stable cell lines were induced in the presence of RA as in (A) and harvested at 12, 24, 48 or 72 h. Cell lysates were subjected to immunoblotting with anti-Nanog, anti-Oct4, anti-Klf4, anti-SATB1, anti-phospho-SATB1 and anti-GAPDH. (**D**) A schematic representation of the dynamic change of Nanog expression in Figure 6C is shown. (**E**) The F9 stable cell lines were induced as in (C) and quantitative RT-PCR was performed to analyze the transcription level of *Nanog*. Results are from three independent experiments. (**F**) The F9 stable cell lines were induced with RA for 0, 12 or 24h and SATB1 occupancy on *Nanog* locus was documented using ChIP assay. (**G and H**) The F9 stable cell lines were induced as in (C) and quantitative RT-PCR was performed for *Bcl2* and *Nestin*, two differentiation genes. (**I**) A working model for Akt-involved pluripotency/differentiation switch. See Discussion for details. The error bars in (E), (F), (G) and (H) represent mean ± SD from three independent experiments. Student’s t-test was performed between wild-type SATB1 and SATB1S47A groups (*p<0.05; **p<0.01).

Mouse F9 embryocarcinoma cells represent a tractable model to characterize the signaling relay from PI3K/Akt to the pluripotency/differentiation factors [28], [29]. During RA-induced differentiation of mouse F9 cells, Akt activity exhibited a transient increase with a peak at 6 h, which was accompanied with a similar pattern change of its substrates as demonstrated by a phosphorylated Akt substrate antibody (Figure 6A). The expression of Nanog increased transiently, reached a peak at 6 h and declined thereafter (Figure 6A). The phosphorylation of Oct4 reaches a peak at 12 h, whereas the total amount of Oct4 dramatically decreased at the end of RA induction, which implies that Oct4 phosphorylation might contribute to its degradation (Figure 6B). In agreement to the profile of Akt substrates, the phosphorylated SATB1 increased transiently and then reduced in the induction process, although the total amount of SATB1 continuously declined (Figure 6C). The dynamic change of SATB1 phosphorylation was also observed in CPT-induced apoptosis (Figure 2F and 2G), corroborating the idea that SATB1 correlated to the differentiation process via the mechanism of Akt-mediated phosphorylation. These results suggest that Akt activation is coupled to the loss of stemness via the phosphorylation of pluripotency/differentiation factors.

In order to ascertain the regulatory role of SATB1 phosphorylation in F9 cell differentiation and Nanog expression, we established F9 stable cells carrying vector control, Flag-HA-SATB1, Flag-HA-SATB1S47A or Flag-HA-SATB1S47D, respectively (Figure S5A). Under normal growth culture, expression of Nanog, Oct4, Sox2 or Klf4 mainfested no significant difference among these stable cell lines (Figure S5A, S5B and S5C). In the presence of RA, the phosphorylated SATB1 was more stable as shown with anti-phospho-SATB1, and the ability of SATB1S47A to repress Nanog expression was dramatically lower than that of SATB1 or SATB1S47D, which agreed to an idea that phosphorylation was critical for the function and stability of SATB1 (Figure 6C and 6D). In addition, the RNA level of *Nanog* was the highest in the F9 cells integrated with the mutant of SATB1S47A in the late stage of differentiation (Figure 6E). Therefore, it was quite possible that SATB1S47A was functionally inert with respect to wild-type SATB1. Such inability could account for the phenomenon that this mutant was enriched to the *Nanog* locus more than SATB1 or SATB1S47D (Figure 6F). Consistently, Wortmannin and AKTi-1/2, inhibitors of PI3K and Akt, delayed the attenuation of Nanog and Oct4 during RA-induced differentiation of ECCs (Figure S5D). On the other hand, the SATB1S47A mutant failed to efficiently activate expression of two differentiation genes, *Bcl2* and *Nestin*, in the RA-induced differentiation process (Figure 6G and 6H). However, we detected neither a striking difference in Klf4 at transcriptional and translational levels upon exogenous expression of SATB1, nor any SATB1 binding on the Klf4 locus using a ChIP assay, which could be caused by a possible genomic structure change within the Klf4 locus in the F9 cell, a carcinoma cell line (Figure S5E). Taken together, the results indicate that Akt is involved in the differentiation of mouse F9 embryocarcinoma cells through coordinated phosphorylations of pluripotency/differentiation factors.

## Discussion

Here, we demonstrate that Akt phosphorylates SATB1, a chromatin organizer and global regulator for gene expression, thereby keeping it intact and maintaining its inhibitory effects on the expression of *Nanog*. On the other hand, Akt phosphorylates pluripotency factors Oct4 and Klf4, promoting their degradation via the ubiquitin-proteasome system. Moreover, SATB1 binding to Sox2 also relies on PI3K/Akt signaling, which probably disrupts complex formation of pluripotency factors as a consequence of Sox2 sequestration. Taken together, we conclude that Akt-mediated phosphorylation would destroy the balance of pluripotency/differentiation factors, ultimately favoring a differentiation process of embryonal carcinoma cells.

We provide several lines of evidence revealing that SATB1 is a novel substrate of Akt. Moreover, SATB1 phosphorylation is perfectly dependent on IGF-1/PI3K signaling and IGF-1/PI3K/Akt/SATB1 presumably constitutes a novel signal relay axis. It is reported that SATB1 partially co-localizes with PML, and when apoptosis occurs is targeted for the PML body where SATB1 is sumoylated and cleaved by caspase 6 [23]. Our data reveals that Akt-mediated phosphorylation shields SATB1 from apoptotic cleavage, but it remains unknown whether SATB1 cleavage is a cause or a result of apoptosis. Consistently, the phosphorylation-mimetic mutant SATB1S47D is reluctant to be localized to PML body, whereas the mutant SATB1S47A is unstable in several breast cancer cell lines. The unstable mutant SATB1S47A reversibly stimulates Akt activation by a yet-to-be identified mechanism, suggesting that a feedback loop exists between Akt and SATB1 in terms of kinase catalytic activity. A number of molecules including PDK1, mTORC2, PP2A, PTEN and PHLPP may serve as candidates to modulate Akt activation, which could be indirectly regulated by SATB1.

Considering the antagonizing effects of SATB1 on pluripotency factors and the fact that Akt phosphorylates SATB1 and protects it from apoptotic cleavage, we predicted that Akt-mediated phosphorylation would abolish the functions of Oct4 and Klf4. Our data revealed that Akt phosphorylated Oct4 and Klf4 and increased their degradations. It is thus realized that the pluripotency factors Oct4, Sox2 and Klf4 are regulated through the same phosphorylation-dependent mechanism [30], [31]. The relationship between IGF1/Akt pathway and Klf4 abundance has already been established, but whether Akt would directly phosphorylate Klf4 was not reported [32]. In agreement with our findings, human Oct4 was shown to be phosphorylated at threonine 234 (corresponding to threonine 228 of mouse Oct4) *in vivo* and a phosphorylation-mimetic mutant in place of wild-type Oct4 significantly reduced the formation efficiency of iPSCs [33]. During RA-induced differentiation of F9 cells, Akt activity increases transiently and then decreases gradually, and the landscape of Akt substrates is perfectly matched to the profile of Akt activity. As examples, phosphorylations of SATB1 and Oct4 manifest the same trend as the dynamic change of Akt activity. On the basis of these observations, it is unlikely that the phosphorylation level of Oct4 is continuously increased in the context of decreased Akt activity and reduced amount of Oct4 [31].

SATB1 transcriptionally represses expressions of Nanog and Klf4 through direct binding on their MAR elements and stimulates expressions of differentiation genes *Nestin* and *Bcl2* during RA-induced differentiation of ESCs [15]. To investigate effects of SATB1 phosphorylation by Akt, we first employed stable F9 mouse embryocarcinoma cell lines and reiterated the above-mentioned effects of SATB1 in ESCs, demonstrating that the F9 cell was an excellent model to elucidate the stimulatory role of SATB1 on differentiation. Importantly, we also observed that the non-phosphorylable mimetic mutant SATB1S47A could not suppress expression of *Nanog*, and that this mutant failed to stimulate the transcriptions of differentiation genes *Bcl2* and *Nestin*. Moreover, PI3K and Akt inhibitors phenocopied the effects of SATB1S47A on the down-regulation of Nanog and Oct4. The serine 47 localizes outside of the MD and Homeobox domains that are required for MAR sequence binding; the phosphorylation of which might modulate the capability of SATB1 to recruit other regulatory proteins or complexes without affecting DNA binding. Unexpectedly, we find that the SATB1S47A mutant is enriched on the *Nanog* locus more than SATB1 and SATB1S47D. This result implies that the phosphorylated SATB1 is more efficient to regulate gene expression than the unphosphorylated form. Interestingly, SATB1 binding to Sox2 also depends on the PI3K/Akt signaling pathway. As a result, such a sequestration probably disrupts the formation of Oct4/Sox2 complexes and compromises their function. Collectively, SATB1 phosphorylation by Akt is pivotal for its role to antagonize the pluripotency factors at both transcription and protein-protein interaction levels.

PI3K and Akt kinases are critical nodes of IGF-1/Insulin-initiated signaling transduction pathway. The complexity of Akt signal and limitations of experimental approaches make it hard to define the precise role of Akt in the control of stem cell homeostasis, i.e. to maintain the stemness or to facilitate the differentiation process. Employing a linear and logic study by establishing kinase/substrates relationship would conceptualize the role of Akt in the context of stemness regulation and is of great importance. Among Akt substrates identified in this study, SATB1 and Oct4/Klf4 represent examples that antagonize each other to keep the homeostasis of stem cells in check. Highly activated Akt would change the opponency by favoring the differentiation factor SATB1, thereby helping set up a threshold for differentiation process (Figure 6G). Our results support the conclusion that Akt is involved in the differentiation of ECCs and is not required for the stemness. According to this model, it is very likely that the efficiency to generate the iPSCs will be low if the cell derived from a tissue that has high endogenous Akt activity is utilized in reprogramming. In adult stem cells residing in the physiological niche, Akt activity is much higher in cycling stem cells than in quiescent stem cells [26]. The cycling stem cells that have experienced a transient proliferation as a result of Akt activation might partially lose the stemness and are ready to enter a differentiation process. Highly-activated Akt, by disrupting the balance established by a set of adult stem cell-specific regulators, could hinder cycling stem cells that are reverting to a state of dormancy. It is only recently that CSCs are proved to exist in tumor by using lineage tracing [34], [35], [36]. CSCs might abduct the mechanism by which normal stem cells use to maintain self-renewal and differentiation potential and are regarded as a source of metastasis and relapse [37]. Considering the dilemma of the general concept that highly activated Akt promotes tumorigenesis and our data implying that low Akt activity maintains the homeostasis of CSCs, it should be beneficial to apply Akt inhibitors to limit Akt activity under control in cancer treatment.

## Supporting Information

Figure S1 ***In vitro*** **kinase assay.** **(A)** GST-fused proteins were purified and subjected to *in vitro* kinase assay. In reactions with γ-^32^P-ATP, autoradiography showed that Akt phosphorylated MBD3, CHD4, Twist1, Bmi1 and Fbxw7. In a parallel experiment using cold ATP, anti-phospho-Akt substrate recognized Twist1 and Fbxw7 in the presence of ATP, but not in the absence of ATP, which was consistent with autoradiogram, a suggestive of specific recognition for phosphorylated proteins. However, regardless the presence or absence of ATP, the antibody recognized GST-Rnf4, indicating a non-specific recognition. Therefore, it is better to combine these two approaches to confirm the phospho-transfer process on a candidate Akt substrate. **(B)** Akt phosphorylates SATB1 at serine 47 *in vitro*. **(C)** Oct4 and Klf4 are candidate Akt substrates. **(D)** Akt phosphorylates Oct4 at threonine 228 and Klf4 at threonine 399. W/O, without; CBB, Coomassie brilliant blue.
(TIF)

Figure S2 **Akt phosphorylates SATB1. (A)** The putative Akt phosphorylation motif on SATB1 is highly conserved among multiple species. A consensus Akt phosphorylation sequence is denoted for comparison. **(B)** A schematic representation of GST-fused SATB1 constructs is shown. **(C)** Phosphorylation of GST-SATB1 1–204 is Akt- and ATP-dependent in the *in vitro* kinase assay. **(D)** A schematic representation for wild-type and mutant SATB1 is shown. **(E)** Akt (Myr) robustly phosphorylates Myc-SATB1, compared to wild-type Akt or Akt (DN). Myc-SATB1 was co-expressed with Myc-Akt (WT), Myc-Akt (Myr) or Myc-Akt (DN) in HEK293T cells and immunoprecipitated Myc-SATB1 was immunobloted with anti-phospho-Akt substrate. **(F)** Akt phosphorylates wild-type SATB1 other than its mutants at serine 47. Myc-SATB1 or its mutants was ectopically expressed together with Akt (Myr) in HEK293T cells. The cell lysates were subjected to immunoblotting with anti-phospho-Akt substrate and anti-Myc. **(G)** Immunoprecipitation with anti-Myc followed by immunobloting with anti-phospho-Akt substrate reveals SATB1 phosphorylation at serine 47 by Akt. **(H)** Reciprocal immunoprecipitation indicated that SATB1, SATB1S557A or SATB1S557D, but not SATB1S47A or SATB1S47D existed in anti-phospho-Akt substrate immunoprecipitates.
(TIF)

Figure S3 **Characterization of antibody against phosphorylated SATB1.** **(A)** Serum of G5647 recognizes SATB1. HEK293T cells were transfected with Myc-tagged SATB1 and cell lysates were subjected with preimmune serum or serum of G5647 from immuned rabbit. **(B)** Serum of G5648 recognizes SATB1. **(C)** Purified anti-phospho-SATB1 (S47) recognizes SATB1. **(D)** The antibody from serum of G5647 recognizes SATB1, but not its mutants of SATB1S47A or SATB1S47D. Myc-SATB1, Myc-SATB1S47A or Myc-SATB1S47D was transfected together with Myc-Akt (Myr) into HEK293T cells, respectively. Immunoprecipitate with anti-Myc or anti-phospho-SATB1 (S47) was subjected to immunoblotting with anti-Myc and anti-phospho-SATB1 (S47), respectively.
(TIF)

Figure S4 **Akt associates with Oct4.** **(A)** A schematic representation of GST-fused Oct4 constructs is indicated. **(B)** Akt phosphorylates all GST fusion Oct4 proteins that contain the predicted Akt phosphorylation motif. **(C)** Both Wwp2 and Trim24 promote degradation of Oct4 and Sox2, but not Klf4. **(D)** Flag-GFP-Oct4 was transfected to HEK293A cells together with Myc-Akt (WT), Myc-Akt (Myr) or Myc-Akt (DN), respectively. The cell lysates were subjected to immunoblotting with anti-Flag, anti-Myc and anti-GAPDH. **(E)** Akt interacts with Oct4. HEK293T cells were co-transfected with Myc-Akt (WT), Myc-Akt (Myr) or Myc-Akt (DN) together with Flag-GFP-Oct4. Immunoprecipitates were subjected to Western blots with anti-Flag and anti-Myc, respectively. **(F)** A reciprocal immunoprecipitation was performed in HEK293T cells transfected as in (E). **(G)** Oct4 binds Akt. GST and GST-Oct4 was purified and utilized to pull-down Myc-tagged Akt. **(H)** A reciprocal GST pull-down as in (G). **(I and J)** Endogenous Oct4 associates with intrinsic Akt. Endogenous Oct4 or Akt was immunopercipitated from whole cell lysates of AB2.2 mouse ES cells. Immunoprecipitates were subjected to immunobloting with anti-Akt1 or anti-Oct4.
(TIF)

Figure S5 **Akt activation correlates to differentiation initiation of F9 cells.** **(A)** Characterization of stable F9 cell lines carrying SATB1 or its mutants. Flag-HA-SATB1 or its mutants was introduced into F9 cells via lentivirus-mediated gene transfer. The cell lysates were subjected to immunobloting as indicated. **(B and C)** Quantitative RT-PCR analysis for the transcription levels of *Oct4* and *Sox2*. The F9 stable cell lines were induced as in Figure 6C. Results were expressed relative to the transcription level of *GAPDH*. **(D)** F9 cells were seeded on petri dishes, induced with RA (1 µM) in the presence of Wortmannin (100 nM) or AKTi-1/2 (200 nM) and harvested at 72 h. Cell lysates were subjected to immunoblotting with antibodies of anti-SATB1, anti-SATB2, anti-Klf4, anti-Sox2, anti-Oct4, anti-Nanog and anti-GAPDH. **(E)** The F9 stable cell lines were induced as in Figure 6C and SATB1 occupancy on *Klf4* and *Bcl2* loci was documented using ChIP assay.
(TIF)

Table S1 **A summary of** ***in vitro*** **kinase assay for candidate Akt substrates.** MS, Mass Spectrometry; IVKA, in vitro kinase assay. ND, not done.
(TIF)

Text S1 **Oligonucleotides used in this study**. The primers for constructs, real-time PCR and ChIP are shown in supplementary information.
(DOC)
