# Supplementary figures and images for: Akt-Signal Integration Is Involved in the Differentiation of Embryonal Carcinoma Cells
Source: PLoS One. 2013 Jun 6;8(6):e64877. doi: 10.1371/journal.pone.0064877 (PMC3675137; doi:10.1371/journal.pone.0064877)

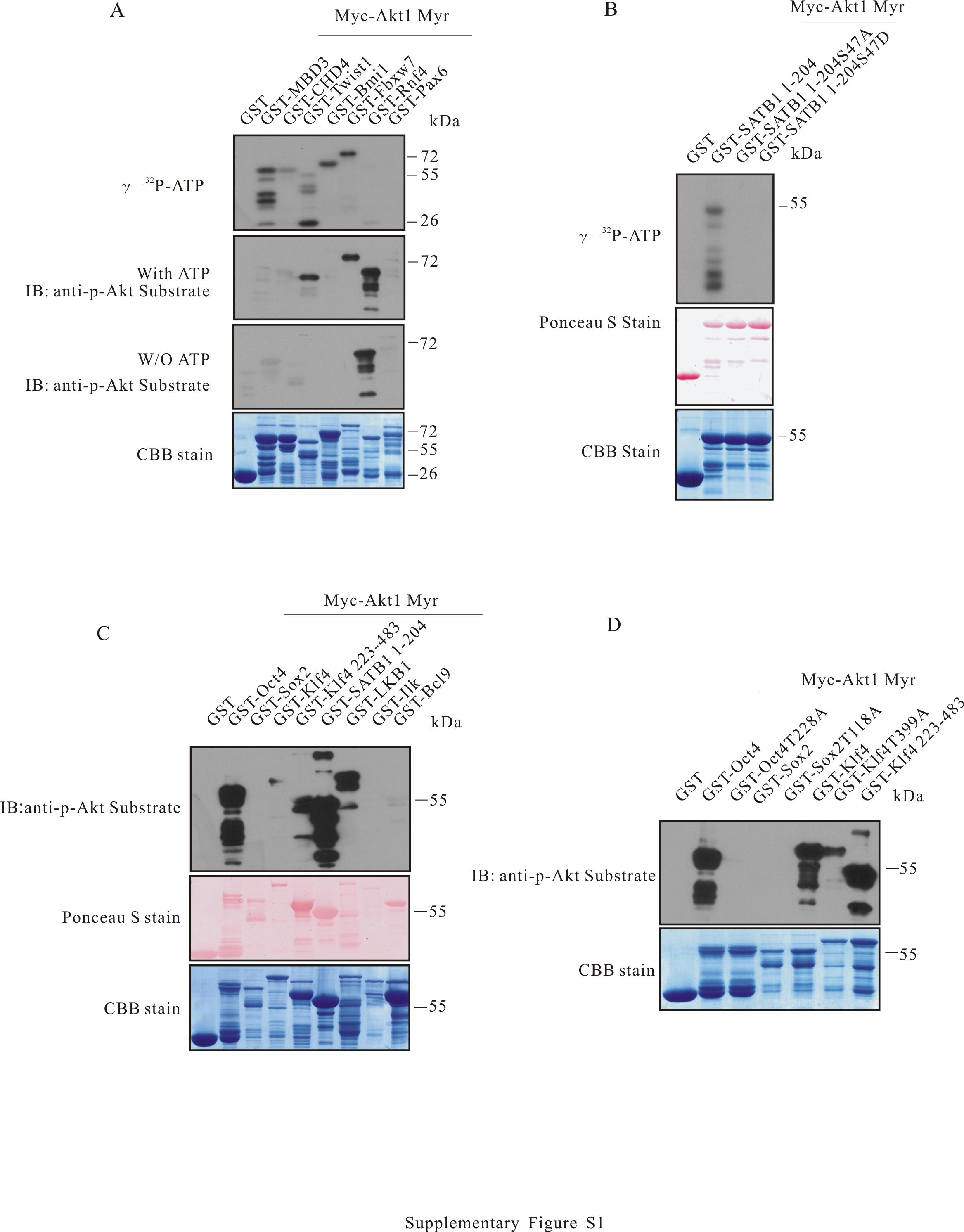

Supplement: Figure S1 — In vitro kinase assay. (A) GST-fused proteins were purified and subjected to in vitro kinase assay. In reactions with γ-32P-ATP, autoradiography showed that Akt phosphorylated MBD3, CHD4, Twist1, Bmi1 and Fbxw7. In a parallel experiment using cold ATP, anti-phospho-Akt substrate recognized Twist1 and Fbxw7 in the presence of ATP, but not in the absence of ATP, which was consistent with autoradiogram, a suggestive of specific recognition for phosphorylated proteins. However, regardless the presence or absence of ATP, the antibody recognized GST-Rnf4, indicating a non-specific recognition. Therefore, it is better to combine these two approaches to confirm the phospho-transfer process on a candidate Akt substrate. (B) Akt phosphorylates SATB1 at serine 47 in vitro. (C) Oct4 and Klf4 are candidate Akt substrates. (D) Akt phosphorylates Oct4 at threonine 228 and Klf4 at threonine 399. W/O, without; CBB, Coomassie brilliant blue. (TIF) [file pone.0064877.s001.tif]

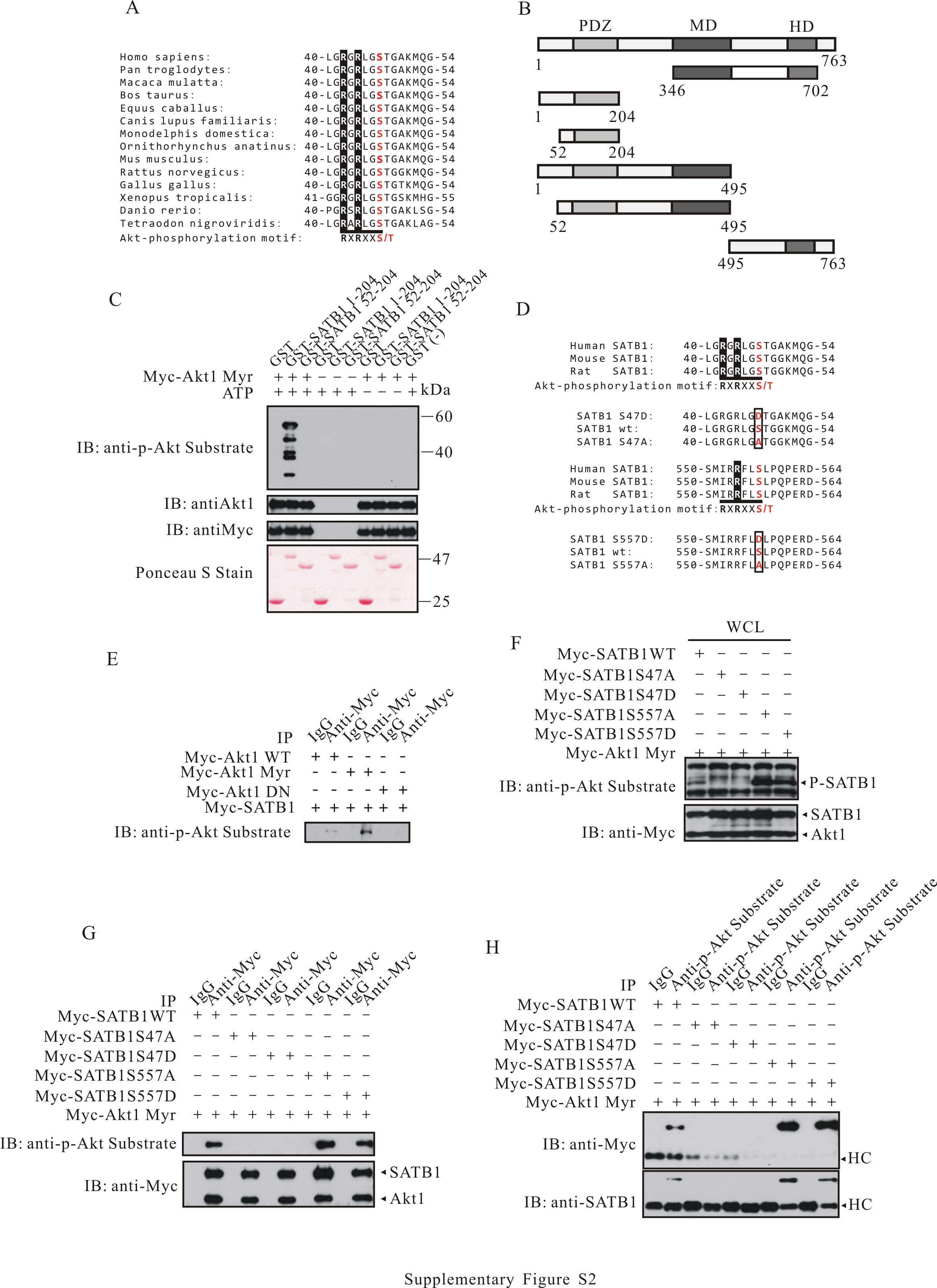

Supplement: Figure S2 — Akt phosphorylates SATB1. (A) The putative Akt phosphorylation motif on SATB1 is highly conserved among multiple species. A consensus Akt phosphorylation sequence is denoted for comparison. (B) A schematic representation of GST-fused SATB1 constructs is shown. (C) Phosphorylation of GST-SATB1 1–204 is Akt- and ATP-dependent in the in vitro kinase assay. (D) A schematic representation for wild-type and mutant SATB1 is shown. (E) Akt (Myr) robustly phosphorylates Myc-SATB1, compared to wild-type Akt or Akt (DN). Myc-SATB1 was co-expressed with Myc-Akt (WT), Myc-Akt (Myr) or Myc-Akt (DN) in HEK293T cells and immunoprecipitated Myc-SATB1 was immunobloted with anti-phospho-Akt substrate. (F) Akt phosphorylates wild-type SATB1 other than its mutants at serine 47. Myc-SATB1 or its mutants was ectopically expressed together with Akt (Myr) in HEK293T cells. The cell lysates were subjected to immunoblotting with anti-phospho-Akt substrate and anti-Myc. (G) Immunoprecipitation with anti-Myc followed by immunobloting with anti-phospho-Akt substrate reveals SATB1 phosphorylation at serine 47 by Akt. (H) Reciprocal immunoprecipitation indicated that SATB1, SATB1S557A or SATB1S557D, but not SATB1S47A or SATB1S47D existed in anti-phospho-Akt substrate immunoprecipitates. (TIF) [file pone.0064877.s002.tif]

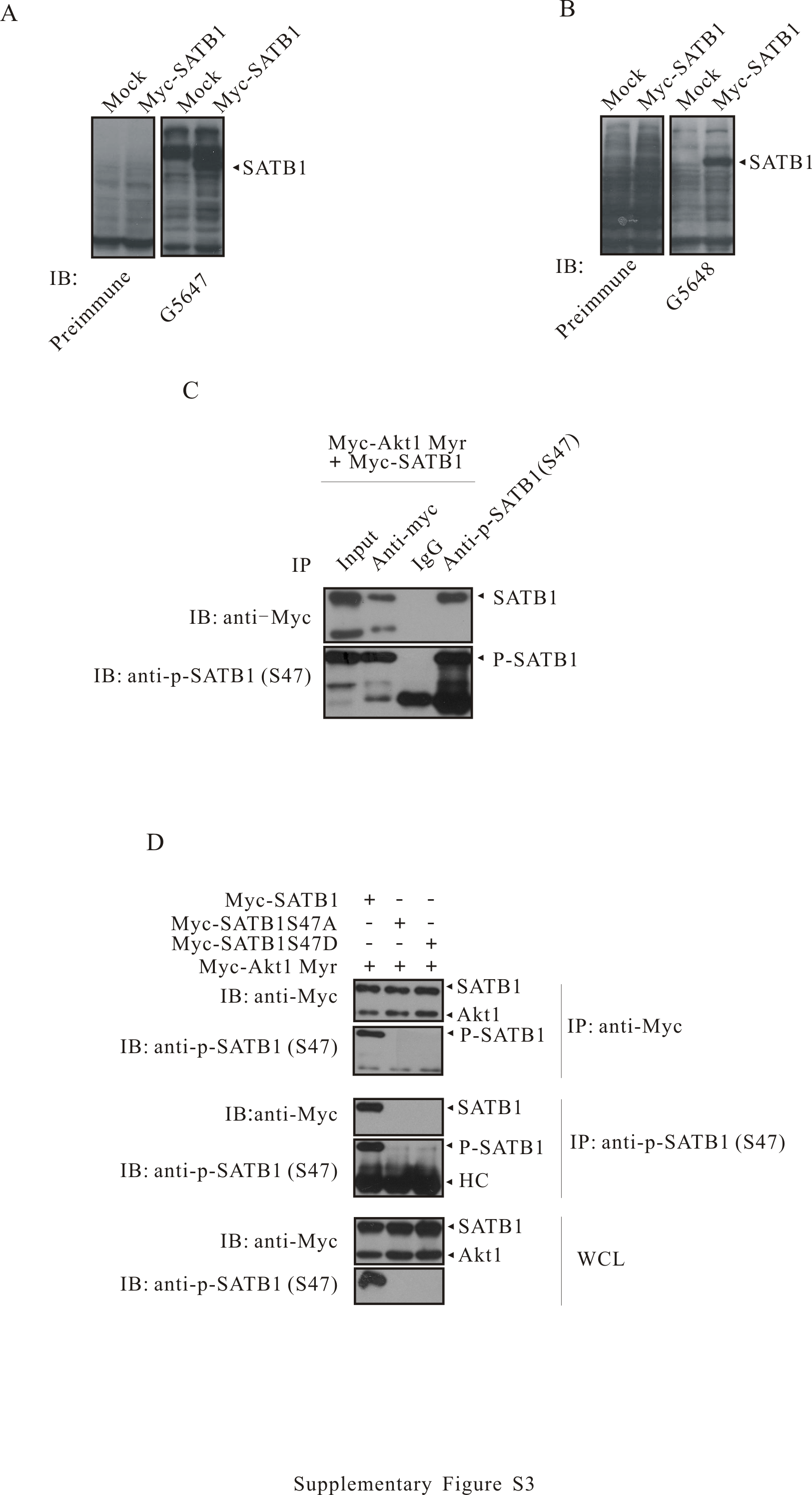

Supplement: Figure S3 — Characterization of antibody against phosphorylated SATB1. (A) Serum of G5647 recognizes SATB1. HEK293T cells were transfected with Myc-tagged SATB1 and cell lysates were subjected with preimmune serum or serum of G5647 from immuned rabbit. (B) Serum of G5648 recognizes SATB1. (C) Purified anti-phospho-SATB1 (S47) recognizes SATB1. (D) The antibody from serum of G5647 recognizes SATB1, but not its mutants of SATB1S47A or SATB1S47D. Myc-SATB1, Myc-SATB1S47A or Myc-SATB1S47D was transfected together with Myc-Akt (Myr) into HEK293T cells, respectively. Immunoprecipitate with anti-Myc or anti-phospho-SATB1 (S47) was subjected to immunoblotting with anti-Myc and anti-phospho-SATB1 (S47), respectively. (TIF) [file pone.0064877.s003.tif]

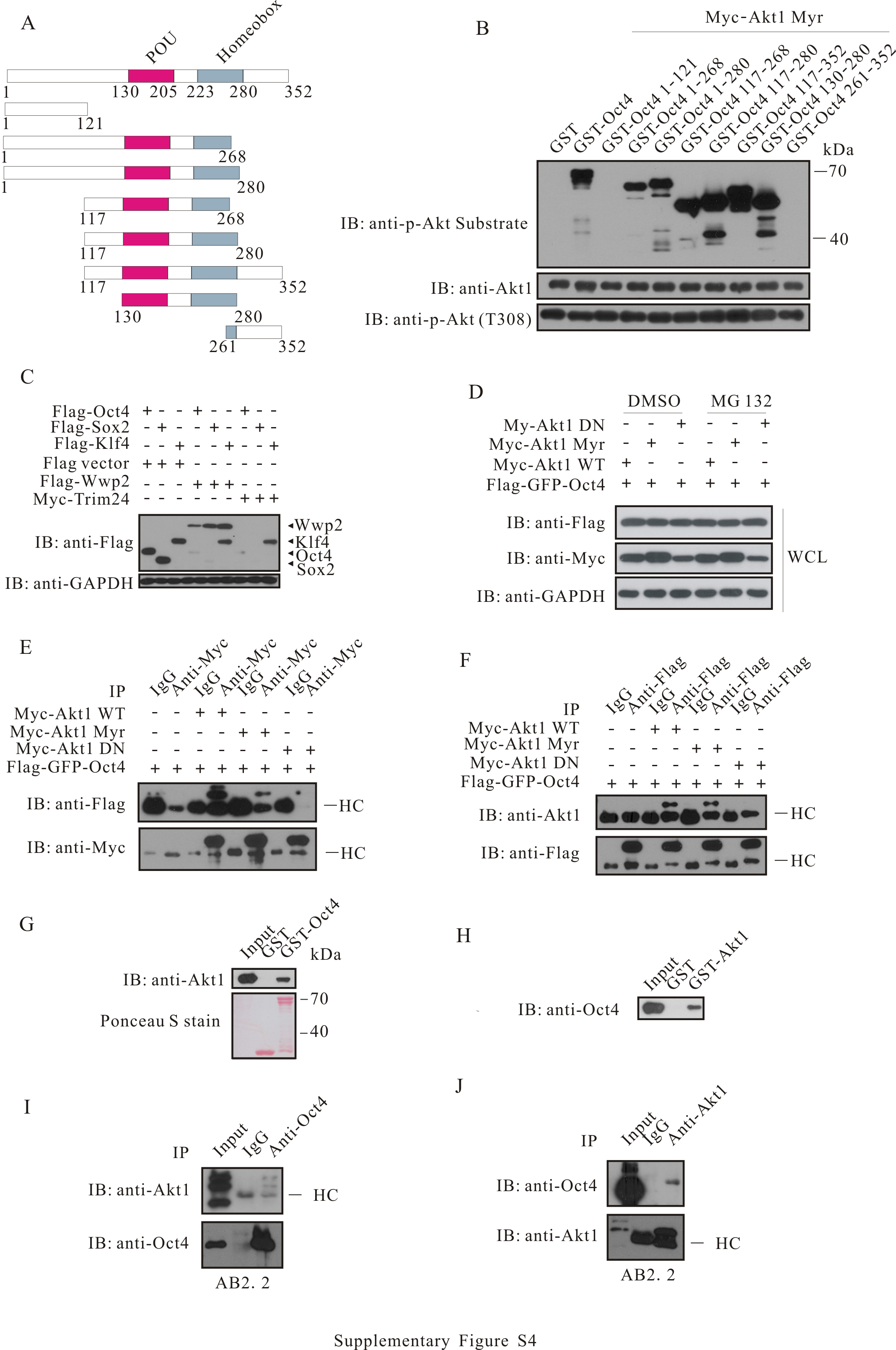

Supplement: Figure S4 — Akt associates with Oct4. (A) A schematic representation of GST-fused Oct4 constructs is indicated. (B) Akt phosphorylates all GST fusion Oct4 proteins that contain the predicted Akt phosphorylation motif. (C) Both Wwp2 and Trim24 promote degradation of Oct4 and Sox2, but not Klf4. (D) Flag-GFP-Oct4 was transfected to HEK293A cells together with Myc-Akt (WT), Myc-Akt (Myr) or Myc-Akt (DN), respectively. The cell lysates were subjected to immunoblotting with anti-Flag, anti-Myc and anti-GAPDH. (E) Akt interacts with Oct4. HEK293T cells were co-transfected with Myc-Akt (WT), Myc-Akt (Myr) or Myc-Akt (DN) together with Flag-GFP-Oct4. Immunoprecipitates were subjected to Western blots with anti-Flag and anti-Myc, respectively. (F) A reciprocal immunoprecipitation was performed in HEK293T cells transfected as in (E). (G) Oct4 binds Akt. GST and GST-Oct4 was purified and utilized to pull-down Myc-tagged Akt. (H) A reciprocal GST pull-down as in (G). (I and J) Endogenous Oct4 associates with intrinsic Akt. Endogenous Oct4 or Akt was immunopercipitated from whole cell lysates of AB2.2 mouse ES cells. Immunoprecipitates were subjected to immunobloting with anti-Akt1 or anti-Oct4. (TIF) [file pone.0064877.s004.tif]

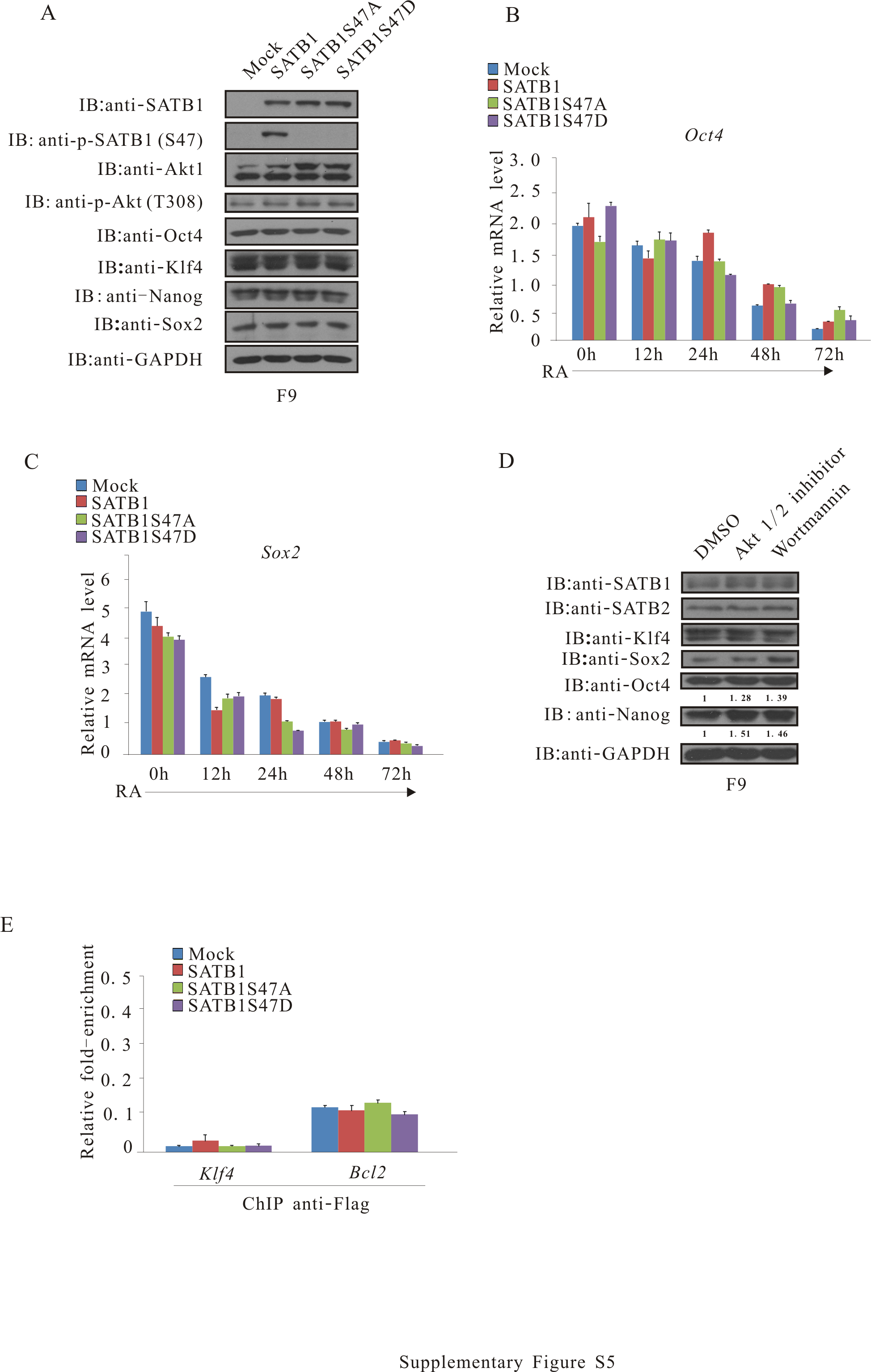

Supplement: Figure S5 — Akt activation correlates to differentiation initiation of F9 cells. (A) Characterization of stable F9 cell lines carrying SATB1 or its mutants. Flag-HA-SATB1 or its mutants was introduced into F9 cells via lentivirus-mediated gene transfer. The cell lysates were subjected to immunobloting as indicated. (B and C) Quantitative RT-PCR analysis for the transcription levels of Oct4 and Sox2. The F9 stable cell lines were induced as in Figure 6C. Results were expressed relative to the transcription level of GAPDH. (D) F9 cells were seeded on petri dishes, induced with RA (1 µM) in the presence of Wortmannin (100 nM) or AKTi-1/2 (200 nM) and harvested at 72 h. Cell lysates were subjected to immunoblotting with antibodies of anti-SATB1, anti-SATB2, anti-Klf4, anti-Sox2, anti-Oct4, anti-Nanog and anti-GAPDH. (E) The F9 stable cell lines were induced as in Figure 6C and SATB1 occupancy on Klf4 and Bcl2 loci was documented using ChIP assay. (TIF) [file pone.0064877.s005.tif]

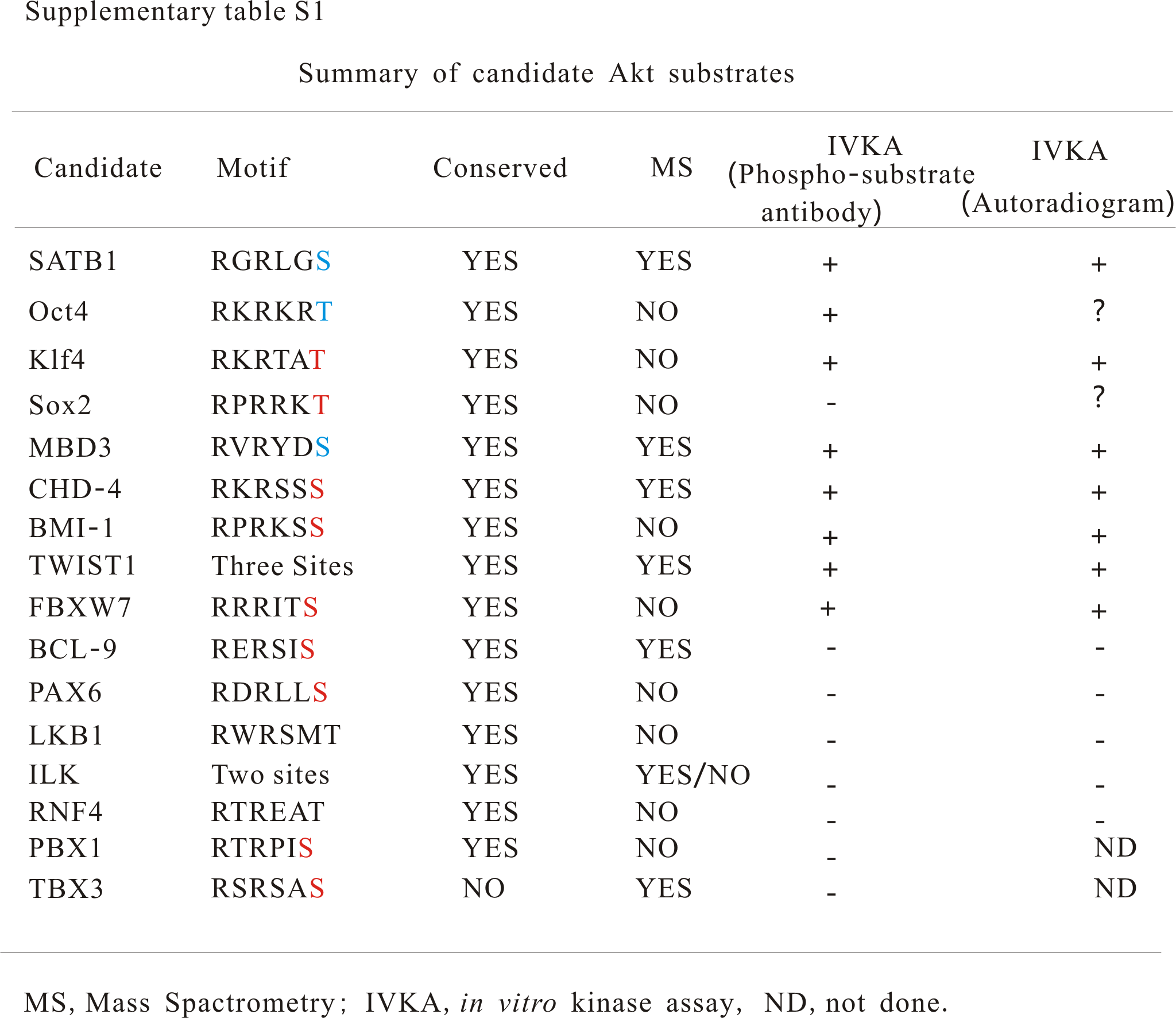

Supplement: Table S1 — A summary of in vitro kinase assay for candidate Akt substrates. MS, Mass Spectrometry; IVKA, in vitro kinase assay. ND, not done. (TIF) [file pone.0064877.s006.tif]
