# Supplementary material for: Akt-Signal Integration Is Involved in the Differentiation of Embryonal Carcinoma Cells
Source: PLoS One. 2013 Jun 6;8(6):e64877. doi: 10.1371/journal.pone.0064877 (PMC3675137; doi:10.1371/journal.pone.0064877)
Supplement: Text S1 — Oligonucleotides used in this study. The primers for constructs, real-time PCR and ChIP are shown in supplementary information. (DOC) [file pone.0064877.s007.doc]

**Supporting Information**

**Primers for Constructs**

Akt1FBamHI 5’-ACGGGGATCCATGAACGACGTAGCCATTGTG-3’

Akt1FEcoRI 5’-ACGGGAATTCATGAACGACGTAGCCATTGTG-3’

Akt1 117RSalI 5’-CGGTCGACGGCCACAGTCTGAATGGCGGT-3’

Akt1 118FEcoRI 5’-ACGGGAATTCGATGGACTCAAGAGGCAGGAA-3’

Akt1 148RSalI 5’-CGGTCGACGTTCATGGTCACACGGTGCTT-3’

Akt1 149FEcoRI 5’-ACGGGAATTCGAGTTTGAGTACCTGAAACTA-3’

Akt1 408RSalI 5’-CGGTCGACAAAGAACCGGTGCTGCATGAT-3’

Akt1 409FEcoRI 5’-ACGGGAATTCGCCAACATCGTGTGGCAGGAT-3’

Akt1 426RSalI 5’-CGGTCGACCTTGAAAGGTGGGCTCAGCTT-3’

Akt1 427FEcoRI 5’-ACGGGAATTCCCCCAGGTCACCTCTGAGACT-3’

Akt2FEcoRI 5’-CGGAATTCATGAATGAGGTGTCTGTCATC-3’

Akt2RXhoI 5’-CGGCTCGAGTCACTCGCGGATGCTGGCCGA-3’

Akt3FEcoRI 5’-CGGAATTCATGAGCGATGTTACCATTGTG-3’

Akt3RXhoI 5’-CGGCTCGAGTTATTCTCGTCCACTTGCAGA-3’

SATB1FEcoRI 5’-CGGAATTCATGGATCATTTGAACGAGGCA-3’

SATB1RXhoI 5’-ACGGCTCGAGTCAGTCTTTCAAATCAGTATT-3’

SATB1S47A

5'-GGAAGAGGAAGGCTTGGGGCTACAGGTGCAAAAATGCA-3'

5'-TGCATTTTTGCACCTGTAGCCCCAAGCCTTCCTCTTCC-3'

SATB1S47D

5'-AGGAAGAGGAAGGCTTGGGGATACAGGTGCAAAAATGCAG-3'

5'-CTGCATTTTTGCACCTGTATCCCCAAGCCTTCCTCTTCCT-3'

SATB1S557A

5'-tgatccgaaggttcctcgctcttcctcagccagaac-3'

5'-gttctggctgaggaagagcgaggaaccttcggatca-3'

SATB1S557D

5'-atgatccgaaggttcctcgatcttcctcagccagaacg-3'

5'-cgttctggctgaggaagatcgaggaaccttcggatcat-3'

SATB1 90FEcoRI

5’-CGGAATTCGCCGCCATGGATTGCAAGGAGGAGCATGCA-3’

SATB1 160FEcoRI

5’-CGGAATTCGCCGCCATGGTGTATCATGTGGTCACATTG-3’

SATB1 205FEcoRI

5’-CGGAATTCGCCGCCATGTTGGCCAAGGAGTGCCCCCTT-3’

SATB1 224FEcoRI

5’-CGGAATTCGCCGCCATGTACTATGCAAATGTCTCAGCA-3’

SATB1 278FEcoRI

5’-CGGAATTCGCCGCCATGACAGCCGAGCAGCCTCCATCC-3’

SATB1 346FEcoRI

5’-CGGAATTCGCCGCCATGTACTTGAACCACCCTCCCCCT-3’

SATB1 763RSalI 5’-ACGGGTCGACGTCTTTCAAATCAGTATTAAT-3’

SATB1 345RXhoI 5’-ACGGCTCGAGTTGTTGGTTTAAGGACTGCTG-3’

SATB1 495RXhoI 5’-ACGGCTCGAGATTAATGTTCATGGTATTGTT-3’

Oct4FEcoRI 5’-CGGAATTCATGGCTGGACACCTGGCTTCA-3’

Oct4RXhoI 5’-CGGCTCGAGTCAGTTTGAATGCATGGGAGA-3’

Oct4 117FEcoRI 5’-CGGAATTCGTGAAGTTGGAGAAGGTG-3’

Oct4 130FEcoRI 5’-CGGAATTCCAGGACATGAAAGCCCTGCAG-3’

Oct4 261FEcoRI 5’-CGGAATTCGGGCTAGAGAAGGATGTGGTT-3’

Oct4 121RXhoI 5’-CGGCTCGAGCTTCTCCAACTTCACGGC-3’

Oct4 268RXhoI 5’-CGGCTCGAGTCGAACCACATCCTTCTCTAG-3’

Oct4 280RXhoI 5’-CGGCTCGAGTCTTTTGCCCTTCTGGCGCCG-3’

Oct4 129RBglII 5’-ACGGAGATCTGGACTCCTCGGGAGTTGGTTC-3’

Oct4 206FBglII 5’-ACGGAGATCTAACAATGAGAACCTTCAGGAG-3’

Oct4 222RBglII 5’-ACGGAGATCTGGCCTGCACCAGGGTCTCCGA-3’

Oct4 281FBglII 5’-ACGGAGATCTTCAAGTATTGAGTATTCCCAA-3’

Oct4T228A

5'-CCGGAAGAGAAAGCGAGCTAGCATTGAGAACCG-3'

5'-CGGTTCTCAATGCTAGCTCGCTTTCTCTTCCGG-3'

Oct4T228D

5'-GCCCGGAAGAGAAAGCGAGATAGCATTGAGAACCGTGT-3'

5'-ACACGGTTCTCAATGCTATCTCGCTTTCTCTTCCGGGC-3'

Oct4T228E

5'-GCAGGCCCGGAAGAGAAAGCGAGAAAGCATTGAGAACCG-3'

5'-CGGTTCTCAATGCTTTCTCGCTTTCTCTTCCGGGCCTGC-3'

Oct4S229A

5'-CGGAAGAGAAAGCGAACTGCCATTGAGAACCGTGTGAG-3'

5'-CTCACACGGTTCTCAATGGCAGTTCGCTTTCTCTTCCG-3'

Oct4S229D

5'-CCGGAAGAGAAAGCGAACTGACATTGAGAACCGTGTGAGG-3'

5'-CCTCACACGGTTCTCAATGTCAGTTCGCTTTCTCTTCCGG-3'

Sox2FEcoRI 5’-ACGGGAATTCATGTATAACATGATGGAGACG-3’

Sox2RXhoI 5’-CGGCTCGAGTCACATGTGCGACAGGGGCAG-3’

Sox2T118A 5’-TACCGGCCGCGGCGGAAAGCCAAGACGCTCATGAAG-3’

5’-CTTCATGAGCGTCTTGGCTTTCCGCCGCGGCCGGTA-3’

Sox2T118D 5’-TACCGGCCGCGGCGGAAAGACAAGACGCTCATGAAG-3’

5’-CTTCATGAGCGTCTTGTCTTTCCGCCGCGGCCGGTA-3’

Sox2T118E

5’-TACCGGCCGCGGCGGAAAGAAAAGACGCTCATGAAG-3’

5’-CTTCATGAGCGTCTTTTCTTTCCGCCGCGGCCGGTA-3’

Klf4FEcoRI 5’-CGGAATTCATGAGGCAGCCACCTGGCGAG-3’

Klf4RXhoI 5’-CGGCTCGAGTTAAAAGTGCCTCTTCATGTG-3’

Klf4223FEcoRI 5’-ACGGGAATTCATGGGCAAGTTTGTGCTGAAG-3’

Klf4T397A

5'-GGCCCCGGAAAAGAGCAGCCACCCAC-3'

5'-GTGGGTGGCTGCTCTTTTCCGGGGCC-3'

Klf4T397E

5'-GTCGTGGCCCCGGAAAAGAGAAGCCACCCAC-3'

5'-GTGGGTGGCTTCTCTTTTCCGGGGCCACGAC-3'

Klf4T399A

5’-CGGAAAAGAACAGCCGCCCACACTTGTGACTAT-3’

5’-ATAGTCACAAGTGTGGGCGGCTGTTCTTTTCCG-3’

Klf4T399D

5’-CGGAAAAGAACAGCCGACCACACTTGTGACTAT-3’

5’-ATAGTCACAAGTGTGGTCGGCTGTTCTTTTCCG-3’

Klf4T399E

5’-CGGAAAAGAACAGCCGAACACACTTGTGACTAT-3’

5’-ATAGTCACAAGTGTGTTCGGCTGTTCTTTTCCG-3’

Wwp2FEcoRI 5’-ACGGGAATTCATGGCATCTGCCAGCTCCAGC-3’

Wwp2RXhoI 5’-CGGCTCGAGCTACTCCTGTCCGAACCCCTCA-3’

Trim24FHindIII 5’-TAGAAGAAGCTTTTCAGAATC-3’

Trim24RHindIII 5’-GATTCTGAAAAGCTTCTTCTA-3’

MBD3FEcoRI 5’-CGGAATTCATGGAGCGGAAGAGCCCGAGC-3’

MBD3RXhoI 5’-CGGCTCGAGCTAGACGTGCTCCATCTC-3’

Rnf4FEcoRI 5’-CGGAATTCATGAGTACAAGAAAGCGTCGT-3’

Rnf4RXhoI 5’-CGGCTCGAGTATATAAATGGGGTGGTACCG-3’

TwistFEcoRI 5’-CGGAATTCATGATGCAGGACGTGTCCAGC-3’

TwistRXhoI 5’-CGGCTCGAGGTGGGACGCGGACATGGACCA-3’

BMIFBamHI 5’-ACGGGGATCCATGCATCGAACAACGAGAATC-3’

BMIRXhoI 5’-CGGCTCGAGTCAACCAGAAGAAGTTGCTGA-3’

PBX1FEcoRI 5’-CGGAATTCATGGACGAGCAGCCCAGGCTG-3’

PBX1RXhoI 5’-CGGCTCGAGTCAGTTGGAGGTATCAGAGTG-3’

TBX3FBamHI 5’-ACGGGGATCCATGAGCCTCTCCATGAGAGAT-3’

TBX3RXhoI 5’-CGGCTCGAGCTACGGGGACGCGCTGCGGGA-3’

PAX6FBamHI 5’-ACGGGGATCCATGCAGAACAGTCACAGCGGA-3’

PAX6RXhoI 5’-CGGCTCGAGTTACTGTAATCTTGGCCAGTA-3’

BCL9FHindIII 5’-CGGAAGCTTATGCATTCCAGTAACCCTAA-3’

BCL9RSalI 5’-ACGGGTCGACTTAAAACATCATGTTTCCTGG-3’

BCL9FEcoRI 5’-CGGAATTCATGCATTCCAGTAACCCTAA-3’

BCL9RSalI 5’-ACGGGTCGACTTTCGGATCATTCCGAAGGGC-3’

FBXW7FEcoRI 5’-CGGAATTCATGAATCAGGAACTGCTCTCT-3’

FBXW7RSalI 5’-ACGGGTCGACTCACTTCATGTCCACATCAA-3’

FBXW7RSalI 5’-ACGGGTCGACGTGCAATGGTTCATCAATCC-3’

**Primers for quantitative RT-PCR**

GAPDHF 5’-GCACAGTCAAGGCCGAGAAT-3’

GAPDHR 5’-GCCTTCTCCATGGTGGTGAA-3’

NanogF 5’-TTCTTGCTTACAAGGGTCTGC-3’

NanogR 5’-AGAGGAAGGGCGAGGAGA-3’

Oct4F 5’-GTTGGAGAAGGTGGAACCAA-3’

Oct4R 5’-CTCCTTCTGCAGGGCTTTC-3’

Sox2F 5’-CTCCATGACCAGCTCGCAGAC-3’

Sox2R 5’-GAGCTGGCCTCGGACTTGACCACAG-3’

Ngn1F 5’-CCAGCGACACTGAGTCCTG-3’

Ngn1R 5’-CGGGCCATAGGTGAAGTCTT-3’

Mash1F 5’-GCAACCGGGTCAAGTTGGT-3’

Mash1R 5’-GTCGTTGGAGTAGTTGGGGG-3’

Klf4F 5’-GTGCCCCGACTAACCGTTG-3’

Klf4R 5’-GTCGTTGAACTCCTCGGTCT-3’

Sox2F 5’-GCGGAGTGGAAACTTTTGTCC-3’

Sox2R 5’-CGGGAAGCGTGTACTTATCCTT-3’

SATB1F 5’-TGCAAAGGTTGCAGCAACCAAAAGC-3’

SATB1R 5’-AACATGGATAATGTGGGGCGGCCT-3’

Bcl2F 5’-GTACCTGAACCGGCATCTG-3’

Bcl2R 5’-GGGGCCATATAGTTCCACAA-3’

NestinF 5’-GAGCTGGAGCGCGAGTTAGA-3’

NestinR 5’-GCCACTTCCAGACTAAGGGA-3’

**Primers used in ChIP experiments (for quantitative RT-PCR)**

Klf4F 5’-TCGCCTGTAAAGGGAAAGAG-3’

Klf4R 5’-GGGGGTGGCTAATTCTTCC-3’

NanogF 5’-CACCCACTTGTTGGCTTGTA-3’

NanogR 5’-AGAAGCCAGAAGAAGGCATCTA-3’

Oct4F 5’-GGGGTGAGAGGACCTTGAA-3’

Oct4R 5’-GGACAGGACAACCCTTAGGAC-3’

Bcl2F 5’-CTGCTTTTTATTTCATGAGGTACATT-3’

Bcl2R 5’-GCCTCTTCACCTTTCAGCAT-3’
